# Setting Goals and Accepting Challenges for Behavior Change—Analysis of Participants’ Interactions With a Digital Multiple Health Behavior Intervention: Mixed Methods Study

**DOI:** 10.2196/66208

**Published:** 2025-08-29

**Authors:** Katarina Åsberg, Marie Löf, Marcus Bendtsen

**Affiliations:** 1 Division of Society and Health, Department of Health, Medicine and Caring Sciences, Linköping University, Linköping, 58183, Sweden, 46 13281000; 2 Department of Medicine Huddinge, Karolinska Institutet, Stockholm, Sweden

**Keywords:** digital intervention, mHealth, mobile health, public health, fidelity, multiple health behavior change, behavioral challenges, goal-setting, action-planning, coping-planning, factorial randomized controlled trial, college and university students

## Abstract

**Background:**

Digital interventions are effective in promoting healthy behaviors and are recognized as one of many strategies for achieving healthier populations. These interventions often include goal-setting, but the practical application and fidelity of goal setting, especially when targeting multiple health behaviors, remain underexplored. In a factorial randomized trial, we included goal-setting as one of six behavior change components in the digital intervention “Buddy,” targeting university and college students’ alcohol, diet, physical activity, and smoking behaviors. However, we found no strong and consistent evidence of an effect of goal-setting alone on any of the outcomes, highlighting the need to investigate how participants used this component.

**Objective:**

This case study of Buddy aimed to gain insight into participants’ interactions with the goal-setting component. Specific objectives were to identify the characteristics of participants who used this component and to analyze participants’ self-authored content.

**Methods:**

This study combined fidelity and effectiveness findings and involved 1704 participants from 18 universities and colleges in Sweden. Self-authored goals and challenges were analyzed using summative content analysis. Logistic and negative binomial regression analyses were conducted to estimate the odds of setting a goal, selecting or self-authoring a challenge, to estimate the odds of setting a goal with respect to a specific behavior, and to estimate the frequency of selecting or self-authoring different behavioral challenges.

**Results:**

Of the 850 participants given access to the goal setting and challenges component, 427 (50%) set at least one goal and 403 (47%) selected or self-authored at least one challenge. A total of 607 goals were set, with most participants setting one goal (336/427, 79%). Goals primarily targeted physical activity (n=302), dietary behavior (n=140), and multiple health behaviors (n=53), typically combining physical activity with diet, alcohol, smoking, or sleep. Other goals included study performance, mental health, sleep, and mobile phone use (n=73). Fewer goals concerned alcohol (n=19) or tobacco (n=17). Participants selected 1506 challenges from 41 premade challenges, with dietary behavior challenges being most popular (667/1506, 44%). An additional 170 challenges were self-authored. Participants’ baseline characteristics were associated with the odds of setting goals targeting specific behaviors and the frequency of selecting or self-authoring challenges targeting specific behaviors.

**Conclusions:**

Our analyses suggest that, while goal-setting is theoretically grounded, and participants used Buddy in ways that suited their personal needs, this did not translate to measurable behavior change in the study population. The self-authored content showed how participants used the component and provided insights into how they articulate behavior change in terms of personal goals, challenges, strategies for action, motivation plans, and rewards. Future research should explore the conditions under which goal-setting may be more or less effective, to better understand its nuances and potential benefits.

## Introduction

Improving unhealthy behaviors using digital interventions is one of many potential public health strategies for achieving healthier populations. Behavioral changes can reduce the incidence and burden of noncommunicable diseases, including cancers, cardiovascular diseases, and diabetes [1-5]. Individual-level public health interventions can support the adoption of healthy behaviors, including reducing alcohol consumption, eating a healthy diet, engaging in regular physical activity, and smoking cessation. In this context, digital interventions have proven effective in facilitating behavior change across various health behaviors [6-13]. Digital interventions offer scalable and easily accessible support designed to strengthen individual skills and capabilities, for example, by guiding individuals in setting goals for behavior change. However, few digital interventions have addressed multiple health behaviors simultaneously, despite the frequent co-occurrence of unhealthy behaviors [14,15].

In the context of behavioral interventions, goal-setting is recognized as a key behavior change technique [16]. It is commonly operationalized in interventions to focus on both behavioral goals, such as “go for a walk,” and outcome-oriented goals, such as “lose weight.” The rationale for this approach is grounded in the goal-setting theory developed by Locke and Latham [17,18], which focuses on the motivational aspects of setting goals. The theory emphasizes that specific and challenging goals, coupled with feedback, may lead to increased effort and improved task performance compared to easy, vague, or nonspecific goals, such as “doing one’s best.” The utility of setting goals is dependent on the individual’s commitment to the goal, possession of the necessary skills to attain it, and absence of conflicting goals. Building on this theory, social cognitive theory [19,20] expands the scope of goal-setting by asserting that health behaviors are influenced not only by an individual’s goals but also by their understanding of potential health risks and benefits associated with the behavior, their belief in their capability to perform these behaviors (perceived self-efficacy), their expectations of the outcome of their actions, and the perceived facilitators and barriers.

Complementing these theoretical frameworks, interventional research provides further insight into the efficacy of goal-setting within digital health interventions. Studies suggest that interventions are particularly effective when key intervention components include goal-setting, along with action planning, practicing behavior, behavior substitution, habit formation, and problem-solving [21-27]. For instance, a meta-analysis by McEwan et al [28], which included randomized controlled trials where participants set goals for physical activity, reported a medium-sized positive effect of goal-setting (Cohen *d*=0.55; 95% CI 0.43-0.67). Furthermore, a review by Epton et al [29] evaluated the effects of goal-setting across a range of behavior outcomes, including health-related goals (eg, weight loss), sporting goals (eg, performance in sports), cognitive goals (eg, problem-solving), and educational goals (eg, increasing study time). The review found small but robust effect sizes (Cohen *d*=0.34; 95% CI 0.28-0.41), suggesting that goal-setting can be beneficial in various behavioral contexts beyond health behavior change.

While goal-setting has been extensively researched, most studies focus on single-behavior interventions with clearly defined behavioral targets such as smoking cessation or physical activity. In contrast, this study investigated its application within the context of a digital multiple health behavior change intervention, which has not been thoroughly examined in previous research. This approach allowed us to explore how participants interpreted and used goal-setting when behavioral goals were not predefined by the intervention, offering insights into its real-world implementation. Based on its theoretical grounding and wide use in other health behavior interventions, we included goal-setting as one of six potentially effective components in a multiple health behavior change intervention [30]. This digital intervention, called Buddy, targeted university and college students’ alcohol, diet, physical activity, and smoking behaviors. The intervention was designed to be open and flexible, allowing participants to use its components based on their individual needs. Our findings revealed no strong and consistent evidence of goal-setting alone impacting any of the measured health behaviors (Asberg et al, unpublished data, 2025). When goal-setting was combined with self-monitoring, there was limited evidence of positive effects on alcohol consumption and smoking 2 months postrandomization, but these effects did not persist at later follow-ups.

If goal-setting is to be an effective component of digital interventions, it is essential to ensure that the component is delivered and received by participants as intended. Evaluating this implementation fidelity can help identify any deviations from the protocol, such as when an intervention component is not delivered or used as planned [31]. Deviations may compromise the impact that an intervention may have; thus, identifying them and revising protocols is important. Despite this, the practical application and fidelity of goal-setting within both digital and face-to-face interventions are rarely evaluated [32,33].

Considering that the evidence we collected did not indicate an effect of goal-setting on behavior, and that the use of goal-setting is understudied in digital multiple behavior interventions, we aimed to explore how participants used this component. To investigate this interaction, we conducted a case study of Buddy with the aim of gaining a deeper understanding of participants’ interactions with the goal-setting component of the Buddy intervention. The specific objectives were to identify the characteristics of participants who used this component and to analyze participants’ self-authored content.

## Methods

### Study Design

This study combined quantitative and qualitative data collected from a factorial randomized trial of Buddy, which invited participants from 18 geographically dispersed universities and colleges in Sweden (representing approximately 50% of all) to take part in a behavior change intervention. Participants were recruited through posters, leaflets, email, websites, social media, and via Student Health Care staff. Interested students registered by texting a dedicated number. Students were eligible for inclusion if they were 18 years or older and met at least one of six criteria for unhealthy behaviors, in accordance with national public health guidelines. These included: (1) having consumed 10/15 (woman/man) or more standard drinks of alcohol in the past week (with one standard drink defined as 12 grams of pure alcohol); (2) having consumed 4/5 (woman/man) or more standard drinks of alcohol on a single occasion at least once in the past month; (3) having consumed less than 500 grams of fruit and vegetables on average per day in the past week; (4) having consumed two or more units of sugary drinks in the past week (one unit defined as 33 cl); (5) having spent less than 150 minutes on moderate and vigorous physical activity the past week; or (6) having smoked at least one cigarette in the past week. Students were excluded if they lacked sufficient proficiency in Swedish to complete the registration or did not have access to a mobile phone, as all study information and intervention materials were delivered digitally in Swedish.

The trial was part of the MoBILE (Mobile Health Multiple Lifestyle Behavior Interventions Across the Lifespan) research program [34], which aims to study digital health interventions across the lifespan, and was designed to evaluate the components of Buddy, of which, goal-setting was one. Buddy is based on previous research of digital health interventions among Swedish university students [35-42], on social cognitive models [43,44], and behavior change techniques [16]. For details of the factorial trial and the intervention, please see the protocol [30].

**Figure 1.**
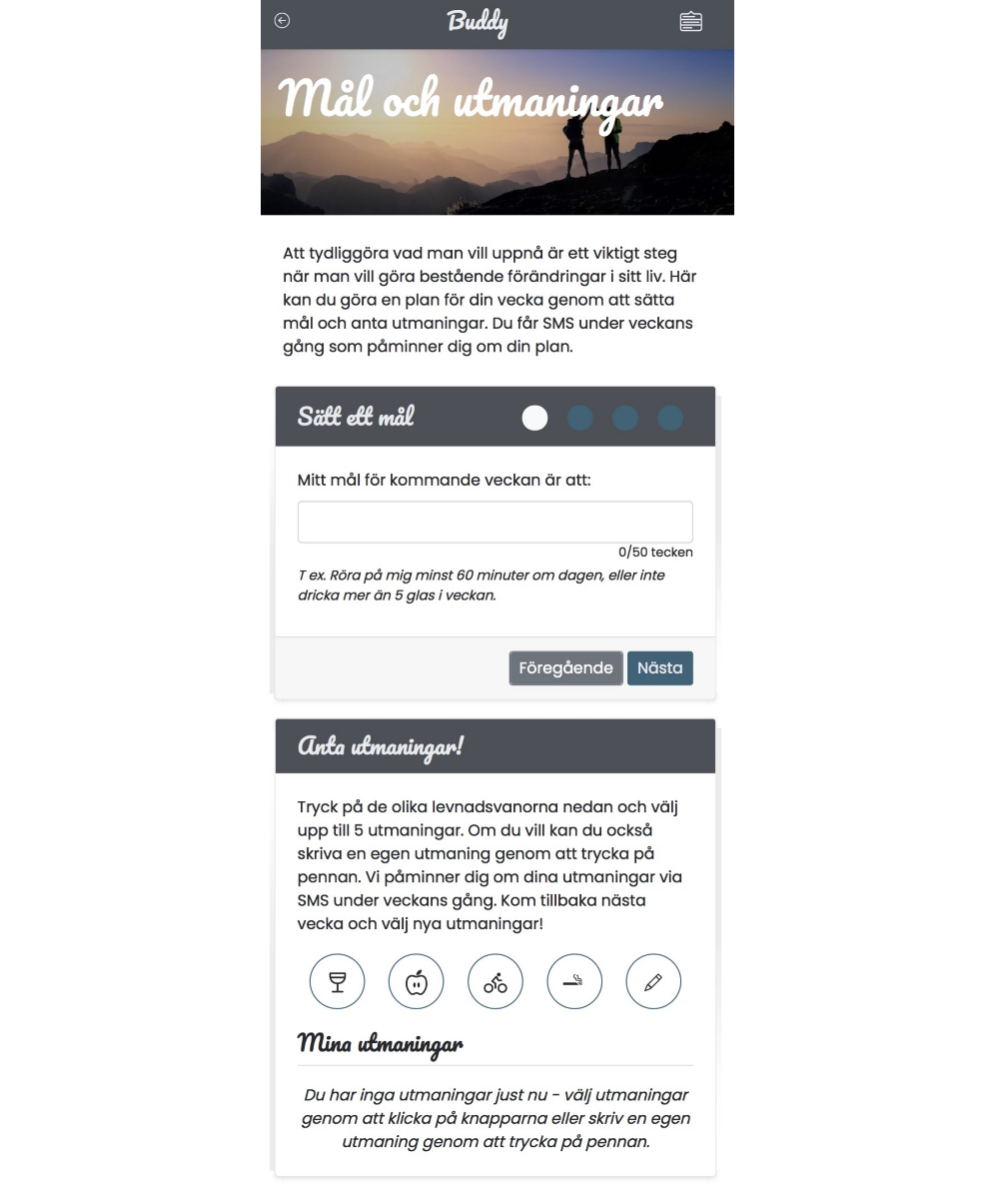
Interface of the goal-setting and challenges module in Buddy, showing where participants could self-author goals for the upcoming week (upper section), and select premade challenges addressing alcohol, diet, physical activity, and smoking. Translation: Goal-setting and challenges. Clearly defining your goals is an important step when aiming to make lasting changes in your life. Here, you can create a weekly plan by setting goals and accepting challenges. Throughout the week, you will receive text message reminders to help you stay on track with your plan. Set a goal. My goal for the upcoming week is to: ...For example, engage in physical activity for at least 60 minutes a day, or limit alcohol consumption to no more than 5 units per week. Accept challenges! Tap on the different health behaviors below and choose up to 5 challenges. If you prefer, you can also create your own challenges by tapping the pencil icon. We will send you text message reminders about your challenges throughout the week. Come back next week to select new challenges!

A screenshot of the goal-setting and challenges component of Buddy is presented in Figure 1. The Buddy intervention aimed to support health behavior change by enhancing self-regulatory skills and capacity through goal-setting and planning. By providing a structured approach, participants were encouraged to set goals and challenges for behavior change in any area they chose and were reminded of these throughout the week via text message. The intervention was accessible to participants over a period of 4 months, with weekly reminders sent on Sundays to access the intervention.

Participants were prompted to set one or more goals for their future behavior, which included: (1) Setting a goal for the upcoming week, participants self-authored what they wanted to achieve; (2) Action-planning, participants formulated two strategies or activities that they planned to use to progress toward their goal; (3) Coping-planning, participants identified and prepared for potential motivational struggles by formulating their approach to self-motivation when faced with barriers; and a (4) Reward-planning, participants selected rewards for themselves upon achieving their goal.

Participants could also create their own challenges or choose from a library of ready-made challenges for the coming week. The challenges were shorter descriptions of tasks, for example, “I will start every day with a 15-minute walk.” Participants were informed that they could return to the system to set new goals and challenges.

### Ethical Considerations

The study received approval from the Swedish Ethical Review Authority on December 15, 2020 (Dnr 2020‐05496). All participants provided informed consent prior to participation. Data were anonymized before analysis to ensure confidentiality. No financial compensation was provided to participants. The trial was preregistered in the ISRCTN registry on January 28, 2021 (ISRCTN23310640).

### Data Analysis

#### Qualitative Analysis

We conducted a qualitative content analysis using a summative approach, as outlined by Hsieh and Shannon [45], to analyze how participants interacted with the goal-setting and challenges component of the Buddy intervention. This approach involved both the quantification of specific content and the interpretation of its contextual meaning, allowing us to explore not only what participants wrote about, but also how they articulated themselves when setting goals and challenges across different health behaviors. The qualitative data consisted of user-generated, self-authored text entries submitted by participants within the intervention. These entries included both self-authored goals and challenges. The self-authored goals were structured entries composed of 5 sentences: one goal formulation, two strategies for achieving the goal, one motivational statement, and one reward. Each goal entry was approximately 60‐80 words long, whereas the self-authored challenges were formulated more succinctly, typically ranging from 2 to 15 words.

All qualitative data were exported from the platform and imported into NVivo12 Plus (Lumivero) for structured coding. We began the qualitative analysis of goals by identifying keywords related to the health behaviors that participants intended to change. Based on these keywords, we grouped the goals according to the targeted behavior, for example, goals related to physical activity were placed in one group, while those related to alcohol use were placed in another. Goals addressing multiple behaviors were categorized independently. Within each behavior group, we then coded the goals by focusing sequentially on content related to strategies, motivations, and finally rewards. This phase involved multiple rounds of coding and iterative refinement through comparing and contrasting codes to ensure attention to detail, accuracy, and consistency. Finally, we developed categories for strategies, motivations, and rewards within each behavior group.

#### Quantitative Analysis

We used logistic regression to estimate the odds of setting a goal and selecting or self-authoring a challenge. We also used logistic regression to estimate the odds of setting a goal with respect to a specific behavior (among those who had set a goal). We used negative binomial regression to estimate the frequency of selecting or self-authoring different behavioral challenges (among those who had selected or self-authored a challenge). All models included baseline characteristics of participants as covariates, including age, sex, BMI, total weekly alcohol consumption, frequency of heavy episodic drinking per month, number of cigarettes smoked per week, minutes of moderate and vigorous physical activity per week, average daily fruit and vegetable intake, weekly sugary drinks consumption, self-perceived stress, and psychosocial measures (importance, confidence, and know-how).

We used Bayesian inference to estimate the parameters of the regression models, with Student *t* priors centered at 0 with 3 degrees of freedom and a scale of 2.5 (a half-Student *t* prior was used for the dispersion parameter of the negative binomial models). We report the median of the posterior distribution as a point estimate of the association, along with 95% compatibility intervals (CI) defined by the 2.5 and 97.5 quartiles of the posterior distribution. We also report on the probability of an association (POA) as the proportion of the posterior distribution that is above or below the null in the direction of the median.

## Results

### Overview

The results are presented in four parts, illustrated in Textbox 1. The first part presents an overview of interactions with the component and the characteristics of the study population. The second part provides a detailed summary of the self-authored goals and challenges. The third part explores the associations between participants’ characteristics and setting goals and challenges, as well as the associations between participants’ characteristics and setting goals and challenges targeting specific behaviors. Finally, the fourth part presents the analysis of participants’ self-authored content regarding goals and challenges.

Textbox 1. **Overview of the four parts in the results**

**Interactions with the component**

- Access to component
- Number of self-authored goals
- Number of self-authored challenges
- Number of selected challenges

**Setting goals and selecting or self-authoring challenges**

- Focus of self-authored goals

- Focus of self-authored challenges

**Associations with participants’ characteristics**

- Participants’ characteristics and setting goals and challenges
- Participants’ characteristics and behavior targeted in goals and challenges

**Participants’ self-authored content**

- Goals
- Strategies
- Motivation
- Reward

### Interactions With the Component and Participant Characteristics

From April 13, 2021, to October 18, 2023, we randomized 1704 participants to the factorial trial. In Table 1, baseline characteristics are presented for the entire study population. Access to the goal-setting and challenges component was given to 850 participants, of whom 427 (50%) set at least one goal and 403 (47%) selected or self-authored at least one challenge. A total of 607 goals were set by 427 participants, the majority of whom set one goal (336/427, 79%), 13% (55/427) set two goals, and 8.4% (36/427) set three or more goals. Additionally, 28 participants started setting a goal but did not fill in the entire form. A total of 1506 challenges were selected from 41 premade challenges, with dietary behavior challenges being the most popular (667/1506, 44%), followed by physical activity (635/1506, 42%), alcohol (154/1506, 10%), and smoking (50/1506, 3%). An additional 170 challenges were self-authored by participants. A complete list of the premade challenges available for selection is provided in Multimedia Appendix 1.

**Table 1.** Baseline characteristics of randomized participants.

Variables	Participants (N=1704)
Sex, n (%)	
Female	1400 (82.16)
Male	304 (17.84)
Age (years), mean (SD)	28 (6.9)
BMI (kg/m^2^), n (%)	
Under (<18.5)	60 (3.52)
Normal (18.5‐24.9)	844 (49.53)
Over (25.0‐29.9)	458 (26.88)
Obese (≥30)	342 (20.07)
Alcohol, mean (SD)	
Total weekly alcohol consumption	3.7 (5.2)
Frequency of heavy episodic drinking	1.8 (2.7)
Smoking	
Number of smokers (smoked ≥1 cigarette per past week), n (%)	218 (12.79)
Number of cigarettes last week, mean (SD)	3.5 (16.6)
Physical activity	
Weekly moderate and vigorous physical activity (minutes), mean (SD)	207 (214)
Dietary behavior, mean (SD)	
Average daily fruit and vegetable consumption, portions per week	1.3 (1.1)
Sugary drinks, cans per week	2.7 (4.1)
Candy and snacks, portions per week	6.5 (6.4)
Stress, mean (SD)	
Self-perceived stress^a^	7.8 (2.9)
Psychosocial measures, median (IQR)	
Importance^b^	8 (7-10)
Confidence^b^	6 (5-8)
Know-how^b^	6 (5-8)

a 4-item perceived stress scale with total score ranging from 0 to 16.

b Single item with 1-10 response options.

### Setting Goals and Selecting or Self-Authoring Challenges

Table 2 shows that most participants set goals for physical activity (n=302) and dietary behavior (n=140). The goals highlighted the interconnectedness of health behaviors as participants set goals targeting multiple behaviors (n=53), typically combining physical activity with other behaviors like diet, alcohol, smoking, and sleep. Participants took the opportunity to set other kinds of behavior goals that focused on a variety of miscellaneous aspects (n=72), such as study-related performance, mental health, sleep, physical body, and mobile phone use. Meanwhile, fewer participants set goals for alcohol (n=19) and tobacco (n=17), including both smoking and snus.

When contrasting the self-authored goals (Table 2) with the self-authored challenges (n=170) in Table 3, similarities emerged regarding the choice of behaviors and activities to be conducted. Challenges, like goals, were primarily about physical activity (n=60), dietary behavior (n=30), mental health (n=19), tobacco use (n=14), study performance (n=12), sleep routines (n=8), leisure activities (n=6), alcohol consumption (n=5), and physical health (n=4). These challenges mirrored the goals’ structure, being articulated as clear directives, intentions, or actions formulated in the first person singular, such as “I shall” or “I shall not.” For example, “I will run every weekday morning this week,” “Eat salad with every meal,” “No smoking before 18.00,” and *“*I will only drink on Friday and Saturday between 19‐22.”

**Table 2.** Focus of self-authored goals.

Focus of self-authored goals	Number of self-authored goals (n=607)
Physical activity	302
Dietary behavior	140
Miscellaneous^a^	72
Multiple health behaviors^b^	53
Alcohol consumption	19
Tobacco including snus	17
Unspecified	4

a Include performance goals, positive mental health goals, sleep behavior goals, physical body goals, and mobile phone use goals.

b Include diet and physical activity goals, combination of behaviors (three or more), physical activity and alcohol goals, physical activity and smoking goals, physical activity and other routine goals, and alcohol or smoking and diet goals.

**Table 3.** Focus of self-authored challenges.

Focus of self-authored challenges	Number of self-authored challenges (n=170)
Physical activity^a^	60
Dietary behavior^b^	30
Mental health: stress reduction, meditation, reflection, gratitude, and self-compassion	19
Tobacco use: reduce and refrain from cigarettes and snus	14
Study performance behavior: time and task commitment for completion	12
Sleeping routines: establish sleep routines	8
Leisure activities: prioritize leisure activities	6
Alcohol consumption: restrictions on consumption	5
Physical health and well-being: prioritize activities for physical health	4

a Number of days exercising per week (n=30), amount of exercise per day (n=16), and specified type of activity (n=14).

b Eat regularly and eat more vegetables (n=23) and reduce snacking (n=19).

### Participant Characteristics and Setting Goals and Challenges

Table S1 in Multimedia Appendix 2 shows associations between baseline characteristics and setting a goal. These analyses reveal that the odds were smaller for men to write a goal compared to women (odds ratio [OR] 0.44, 95% CI 0.30-0.66; POA>99.9%). In addition, those with higher consumption of candy and snacks had higher odds of writing a goal; however, the association did not appear as strong (OR 1.03, 95% CI 1.00-1.05; POA=98%). Those who rated the importance of changing their health behaviors as higher at baseline also had higher odds of setting a goal (OR 1.09, 95% CI 1.00-1.18; POA=97.4%). Finally, although the evidence was weaker, smoking more cigarettes per week at baseline was associated with higher odds of setting a goal (OR 1.12, 95% CI 0.96-1.32; POA=92%).

Table S2 in Multimedia Appendix 2 shows associations between baseline characteristics and selecting or self-authoring a challenge. As was the case for goal-setting, men had a lower odds of selecting challenges compared to women (OR 0.46, 95% CI 0.30-0.67; POA>99.9%). There was also evidence, albeit weaker, that those who rated higher importance of change had higher odds of interacting with challenges (OR 1.07, 95% CI 0.98-1.16; POA=93.6%). Additionally, there was some evidence suggesting that individuals with high BMI (obese) were more likely than individuals with normal BMI to interact with the challenges component (OR 1.32, 95% CI 0.89-1.97; POA=91.4%).

### Participant Characteristics and Behavior Targeted in Goals and Challenges

#### Overview

Tables S3-S16 in Multimedia Appendix 2 show associations between participants’ characteristics and setting goals targeting specific behaviors, and the frequency of selecting or self-authoring challenges targeting specific behaviors. These analyses were done among participants who had set a goal or selected a challenge, respectively. Here, we give a summary of the findings presented in the tables.

#### Man Versus Woman and Age

When comparing men versus women, the strongest associations were found concerning setting goals targeting alcohol and diet, with the odds being lower for men. This pattern was also evident for selecting challenges related to alcohol and diet, where men selected fewer of these compared to women. In addition, men selected fewer challenges related to physical activity than women. Older participants had higher odds of setting goals targeting alcohol consumption and selected fewer challenges relating to diet.

#### Alcohol Consumption

Participants who reported a higher weekly consumption of alcohol at baseline had lower odds of setting goals targeting diet and higher odds of setting goals targeting multiple health behaviors and miscellaneous goals. The multiple health behavior goals concerned combinations of behaviors such as physical activity, diet, alcohol, and smoking, while miscellaneous goals concerned behaviors not specifically targeted by the intervention, such as sleep. Those with higher weekly alcohol consumption also selected more challenges relating to alcohol consumption, but fewer challenges relating to physical activity. More frequent heavy episodic drinking at baseline was associated with selecting more alcohol-related challenges and writing fewer self-authored challenges.

#### Smoking

Participants who reported being more frequent smokers at baseline had higher odds of setting tobacco-related goals and had lower odds of setting goals for all other behaviors (except for multiple health behavior goals). These participants also selected more smoking challenges and fewer diet challenges.

#### Dietary Behavior

Participants with a higher fruit and vegetable intake at baseline had higher odds of setting goals related to physical activity and were less likely to set goals related to alcohol, diet, and tobacco. Similarly, these participants selected more physical activity and smoking challenges, but fewer diet challenges. Participants who reported a higher weekly consumption of sugary drinks had higher odds of setting multiple health behavior goals. Finally, participants who had higher candy and snacks intake at baseline selected more diet and self-authored challenges.

#### BMI

Participants with higher BMI (overweight and obese) had lower odds of setting goals targeting alcohol, as well as selecting fewer alcohol-related challenges, compared to those with normal BMI. Participants in the highest BMI category (obese) had higher odds of setting goals relating to diet and lower odds of setting goals targeting multiple health behaviors. Participants with lower BMI (<18.5) were more likely to set goals targeting diet but selected fewer challenges for diet compared to those with normal BMI. The participants in the underweight category were also less likely to set goals targeting tobacco. Participants in the edge BMI categories (underweight and obese) selected fewer challenges relating to physical activity and smoking, compared to those in the normal BMI category.

#### Physical Activity

Participants with higher levels of physical activity at baseline had higher odds of setting goals targeting diet and miscellaneous goals, and lower odds of setting physical activity goals. They also authored more of their own challenges and selected fewer physical activity challenges.

#### Stress Levels

Participants who experienced higher levels of stress relative to the group mean had higher odds of setting miscellaneous goals and lower odds of setting goals targeting alcohol. These participants also selected more smoking-related challenges.

#### Psychosocial Measures

Participants who rated the importance of changing their behavior as higher relative to the group mean at baseline had higher odds of setting goals targeting alcohol consumption and self-authored fewer challenges. There were no marked associations between confidence in being able to change with goal-setting and challenges. Finally, those who rated their knowledge of how to change their behavior higher relative to the group mean had lower odds of setting goals targeting alcohol and smoking and more frequently self-authored challenges.

### Participants’ Self-Authored Content From Buddy

The content of the self-authored goals and challenges was qualitatively analyzed. The coding process resulted in a total of 2412 codes. They focused primarily on health improvement, behavior modification, and self-improvement. A summary of all self-authored goals, strategies, motivations, rewards, and challenges can be found in Multimedia Appendix 3.

### Participants Articulated Goals Differently Depending on Health Behavior

Goals related to alcohol, tobacco, and physical activity were often quantifiable, focusing on aspects like quantity, frequency, type, or duration. Physical activity goals were typically more specific, detailing activity duration or training frequency. Dietary behavior goals, while also specific, focused more on restrictions, as well as establishing desirable healthy patterns. As expected, considering the complexity of diet, these goals, unlike other health behaviors, included both preventive measures, such as avoiding unhealthy food products, and promotive aspects like incorporating healthy food choices. This contrasted with physical activity goals, which solely promoted increased activity, and alcohol and tobacco goals, which were purely preventive, such as limiting, reducing, or abstaining from the behavior.

*In the upcoming week, my goal is to...*

*...not drink alcohol on weekdays (Alcohol)*

*...not smoke more than 4 cigarettes a day (Tobacco)*

*...go for a walk every morning before studying (Physical activity)*

*...not eat sugar and swap to fruit and berries instead... (Diet)*

*...study at least 4 hours a day (Miscellaneous)*

*...consume 500 grams of vegetables daily and work out 5 times (Multiple)*

### Participants Intend to Use Proactive Strategies Such as Planning and Preparation

When formulating goals, participants were asked to formulate strategies to achieve their goals. Planning, preparation, and prioritization were central to all behaviors and were pronounced as specific, detailed, and proactive strategies on how to achieve goals. This involved setting new routines, being mindful, and focusing on goal-oriented activities.

Participants articulated diverse activities and combinations of strategies. Dietary strategies included meal planning and availability of healthy alternatives, preparation of healthy food, and bringing fruit or lunch boxes to university. Physical activity strategies involved scheduling activities, making room for exercise in one’s study schedule, adjusting routines, setting reminders, and finding motivational tools such as podcasts or social support. Alcohol and tobacco strategies involved heightened awareness of what is important to them and planned modifications to habits, such as slowing drinking pace, not carrying cigarettes, leaving parties earlier, or making environmental changes like avoiding alcohol-related activities.

*In the upcoming week, I will undertake these two actions to progress towards my goals...*

*...buy fruits to eat instead and eat proper breakfast, lunch, and dinner (Diet)*

*...by going to bed on time and making a lunchbox the day before (Physical activity)*

*...make some plans for Saturday night instead of going to the pub (Alcohol)*

*...chew gum, drink tea, and do breathing exercises (Tobacco)*

*...to go to bed on time and not sleep too late during the day (Miscellaneous)*

*...schedule training and say no to cigarettes (Multiple)*

Physical activity strategies were typically action-oriented, specific, and time-framed, closely aligning with the goals. For instance, a goal to walk 20 minutes per day could involve the strategy to go for a morning and a poststudy walk. Conversely, dietary, tobacco, and alcohol strategies involved a broader range of activities like distractions, restrictions, substitutes, and limitations of unhealthy alternatives, as well as planning and preparing for healthy choices. For example, a typical goal to limit drinking involved the strategy to abstain from drinking before going out or swapping to alcohol-free alternatives. A typical goal to avoid unhealthy food involved the strategy to abstain from buying and replacing dessert with fruit and berries instead. Physical activity was also articulated as a strategy to achieve dietary, alcohol, and tobacco goals, such as being physically active as a means to divert attention from snacking, smoking, or drinking.

### Participants Motivate Themselves With Health Benefits, Self-Encouragement, and Goal Focus

When formulating strategies, participants were asked to formulate how to motivate themselves when faced with barriers to achieve their goals. Participants’ motivational self-talk, across various health behaviors, was expressed in a similar manner, primarily focusing on well-being, health benefits, self-encouragement, and goal orientation. The self-talk emphasized both short- and long-term well-being and health benefits. For tobacco, the focus was mainly on the long-term benefits, while physical activity and alcohol were more focused on short-term well-being, such as “feeling better afterwards, or the next day.” Participants recalled and expressed previous experiences of feeling good when adopting a healthy behavior. For example, “I always feel better after exercising” (Physical activity), “I will be feeling so much better if I don’t snack and avoid sweets” (Diet), “This will bring me energy” (Multiple), “I will avoid anxiety the day after if I drink moderately” (Alcohol), “Smoking is harmful to me and my asthma will improve without it” (Tobacco), and “I will feel good from exercise and less screen time. It will help” (Miscellaneous).

Participants used self-encouraging positive affirmations like “you can do it” to motivate themselves. In the context of physical activity, this included pushing oneself to work hard with a just-do-it attitude, overcoming barriers, and reminding themselves of the necessity of effort for success. For diet, tobacco, and alcohol goals, the focus shifted to reminding oneself that there are better alternatives, encouraging perseverance, and strengthening one’s capability to combat craving, all with the aim of making oneself proud. Overall, participants reminded themselves of their capability and determination with affirmations like “I can and I will. They also reassured themselves of their past accomplishments, reinforcing their belief in their capabilities and the manageability of the task.

*If it feels tough, I will tell myself that...*

*...get a grip, it goes quickly and it’s fun! (Physical activity)*

*...I actually prefer and feel better with good, cooked food (Diet)*

*...I feel much better during the week if I don’t drink (Alcohol)*

*...that you cannot afford it, and that I have succeeded in several demanding challenges before (Tobacco)*

*...you can do it, you are goal-oriented and wonderful (Miscellaneous)*

*...you must keep your promises to yourself so that you can trust yourself (Multiple)*

Regarding goal orientation, participants reminded themselves to stay focused on the goal, its significance, and why it is important, for example, the long-term benefits, self-improvement, or success with a task or studies. For miscellaneous behaviors, participants also highlighted the importance of self-value, personal sustainability, and life balance by encouraging themselves to focus on the task and dedicate time for health.

### Participants Plan to Reward Themselves With Shopping, Indulgence, and Self-Appreciation

To meet their goal, participants were asked to articulate how they would reward themselves when achieving their goal. These rewards varied, but overall included shopping, indulging in something to eat, and engaging in enjoyable activities. Regardless of health behavior, participants also planned to express self-appreciation by acknowledging and recognizing their progress and effort. This often took the form of symbolic gestures like “a pat on the back,” applause, a hug, or a sense of pride.

Shopping was a popular reward, with participants planning to buy clothing, beauty products, craft materials, electronics, or home decor. Some participants planned to indulge in culinary treats, such as cooking their favorite meal, dining out, or purchasing “special items” like sushi, exotic fruits, or barista coffee. Among those who set goals related to tobacco and alcohol, some planned to reward themselves with the ability to drink or smoke. Engaging in enjoyable activities was also a common reward. These activities ranged from socializing, movie nights, and gaming to outdoor activities like walking in nature or self-care activities like taking a day off from studying. Some planned to treat themselves with a visit to the hairdresser, a tattoo artist, or a massage, while others planned a trip. Some participants planned to save or invest the money they would have otherwise spent on unhealthy behaviors.

## Discussion

### Principal Findings

We conducted this study to gain insight into the fidelity of a digital multiple lifestyle behavior intervention by investigating participants’ interactions with a goal-setting and challenges component. The self-authored content shed light on how participants used the goal-setting component and provided insights into how participants articulate behavior change in terms of personal goals, challenges, strategies for action, motivation plans, and rewards. Overall, our findings indicate that participants who engaged with the intervention component did so as intended, demonstrating the feasibility of incorporating goal-setting into a multiple health behavior intervention. Despite this, as mentioned earlier, in our analyses of the effects of goal-setting on behavior, we found no strong evidence that the component was effective (Asberg et al, unpublished data, 2025). This may partially be explained by the use being heterogeneous, with many not using the component at all, and individual characteristics determining engagement within the component. However, the combined findings of fidelity and effectiveness suggest that while components of behavioral interventions may be grounded in theory and used as intended, this does not always translate to behavior change. This calls for further research to explore the conditions under which goal-setting may be more or less effective, to better understand its nuances and potential benefits.

Our findings show that participants’ baseline characteristics were associated with setting goals targeting specific behaviors and the frequency of selecting or self-authoring challenges targeting specific health behaviors. These associations were in line with what was anticipated and intended; for instance, participants with higher alcohol consumption focused on alcohol, while smokers prioritized smoking cessation. Meanwhile, despite Buddy’s strong emphasis on alcohol, diet, physical activity, and smoking, those experiencing stress concentrated on goals and challenges concerning managing studies, mental well-being, sleep, and mobile phone use. Our findings also suggest that participants who already practice some healthy behaviors, such as consuming fruits and vegetables or engaging in physical activity, were more inclined to focus on other health areas where they may not be following guidelines. Overall, our findings suggest that participants used the intervention component to address their personal needs for support in changing unhealthy health behaviors.

When analyzing the content of the goals and challenges set by participants, we found that strategies, motivations, and rewards focused on proactive activities like planning, preparation, and prioritization. Participants were motivated by reminding themselves of the health benefits, self-encouragement, and maintaining a focus on their goals. These motivations had both a short- and long-term perspective. Participants supported themselves with positive affirmations, such as “you can do it,” and richer messages of self-improvement, self-worth, and sustainable life balance.

### Comparison With Prior Work

Previous studies have shown that goal-setting can lead to improved outcomes in various health behaviors. For instance, a web-based intervention [46] showed that weekly goal-setting can increase fruit and vegetable consumption among university students over time, but no corresponding impact on physical activity levels was observed. Researchers suggest that while goal-setting can be beneficial in initiating new behaviors, it might not be as effective for enhancing behaviors that individuals are already practicing. In a factorial trial [47], researchers compared adaptive goals versus static goals, and the effect of immediate versus delayed financial rewards on increasing physical activity. The result indicated that static goals initially increased daily step count but subsequently declined physical activity levels over time. Conversely, those with adaptive goals showed a smaller initial increase but maintained a more consistent level of activity throughout the 4-month intervention period. Notably, the activity levels of the static group eventually fell below those of the adaptive goal group, even with the incentive of immediate rewards.

In a study by Padovano et al [48], the relationship between daily goal-setting and its impact on alcohol consumption was investigated among individuals with alcohol use disorder. The study revealed that the most commonly selected goal was complete abstinence (“I have a plan to not drink”; n=858, 31%), followed by moderation goals (“2 or fewer drinks”; n=604, 22%), or (“5 or fewer drinks”; n=618, 22%). The odds of drinking were reduced by 89% when an abstinence goal was set (OR 0.11, 95% CI 0.08-0.15). Additionally, the study observed that those who more frequently set abstinence goals were the ones who drank more per drinking occasion. These formulations align with the self-authored alcohol and tobacco goals in this study, which were framed in a preventive manner—limiting, reducing, or abstaining from the behavior—and were predominantly formulated by participants with higher alcohol and smoking behaviors.

Similarly, the self-authored goals in this study, particularly those related to physical activity, aligned with the self-authored goals set by participants in a digital weight-loss intervention [49]. In that study, participants were encouraged to set weekly behavioral goals and share their goals and accomplishments on a shared social media platform. Findings indicate that goals expressed in repeating terms, or as both measurable and repeating terms (eg*, “*My exercise goal is to walk for at least 15 minutes a day this week”) were associated with greater weight loss. These repeating term goals, for example, articulating the number of training days per week, to eat fruit every day, to meditate, or to study for a specified number of hours per day, were also articulated among the students in this study.

### Limitations

This study has a number of limitations that should be considered. First, associations between baseline characteristics and use of the component are not causal and should not be interpreted as such. These analyses describe who among the participants did what, but not why they did so. Second, the study did not assess whether interaction with the goal-setting component translated into behavior change. To establish causal relationships and better understand the impact of goal-setting on health behaviors, future studies could randomize participants to different goal-setting strategies—such as predefined versus self-authored goals to evaluate their differential effects on behavior change outcomes. Third, half of the participants with access to the goal-setting and challenges component did not choose to engage with it. While we do not expect all participants to interact with digital interventions that have low barriers to entry, it remains an important question to investigate reasons why the remaining participants, despite signing up for Buddy, did not use this module. Future studies could include follow-up with nonusers to better understand barriers to interaction, such as lack of time, perceived relevance, or competing academic or personal demands. Gaining insights into these factors could inform the design of digital interventions that better align with users’ contexts and needs.

Moreover, among those who did engage with the goal-setting component, the majority set one or two goals (n=391, 92%). This could be due to various reasons, including satisfaction with the initial goal or behavior change progress, which might have eliminated the need to set other goals. The process of self-authoring goals may be cognitively demanding, and not all participants are likely to benefit equally from a goal-setting component. Some may find the task of setting and achieving goals difficult, overwhelming, or simply outside the scope of their current priorities. Additionally, the absence of feedback on progress or positive reinforcement from Buddy may have contributed to a lack of motivation to set new goals. Without encouragement to revisit or revise their goals, participants may have disengaged over time.

### Conclusions

Our analyses of self-authored content from the goal-setting and challenges component in a digital intervention for multiple health behavior change provided insights into how participants articulate behavior change in terms of personal goals, challenges, strategies for action, motivation plans, and rewards. Participants set goals related to both single and multiple health behaviors, which is in alignment with the intended purpose of Buddy. Furthermore, participants used Buddy in ways that suited and supported their personal behavior change needs, sometimes extending beyond the scope of the lifestyle behaviors that Buddy targeted. Future research should explore the conditions under which goal-setting may be more or less effective, to better understand its nuances and potential benefits.

## Supplementary material

10.2196/66208 Multimedia Appendix 1 A complete list of the premade challenges available for selection is provided in Appendix 1.

10.2196/66208 Multimedia Appendix 2 Associations between baseline characteristics and setting a goal.

10.2196/66208 Multimedia Appendix 3 A summary of all self-authored goals, strategies, motivations, rewards, and challenges can be found in Appendix 3.

## Abbreviations

CI: compatibility interval
MoBILE: Mobile Health Multiple Lifestyle Behavior Interventions Across the Lifespan
OR: Odds ratio
POA: Probability of an association
