# Supplementary material for: Setting Goals and Accepting Challenges for Behavior Change—Analysis of Participants’ Interactions With a Digital Multiple Health Behavior Intervention: Mixed Methods Study
Source: JMIR Hum Factors. 2025 Aug 29;12:e66208. doi: 10.2196/66208 (PMC12396776; doi:10.2196/66208)
Supplement: Multimedia Appendix 1 [file humanfactors-v12-e66208-s001.docx]

| Most frequently chosen pre-made challenges | Number of challenges  (n=1506) | |
| --- | --- | --- |
| Dietary behavior  I will not buy candy or snacks this week (n=168)  I will eat fruit as a snack every day this week (n=127)  I will replace a bun, piece of chocolate or other candy with fruit this week (n=92)  I will eat vegetables with my main meal every day this week (n=82)  I will completely abstain from soda this week (n=59)  I will google a vegetarian recipe and try it out (n=40)  I will buy and try two fruits, vegetables, or root vegetables that I have not eaten before (n=32)  I will eat breakfast with vegetables every day this week (n=26)  I will halve the amount of energy drink I consume this week (n=21)  I will calculate how much money I spend on soda and energy drinks (n=16)  I will read the ingredient list on three products the next time I shop groceries (n=4) | | 667 |
| Physical activity  I will stand up/move for 3-5 minutes every time I have been sitting for an hour (n=140)  I will go for a 10 minute walk, do 10 squats, or 10 pushups twice this week (n=129)  I will use the stairs every day this week (n=93)  I will start every day with a 15 minute walk (n=76)  I will find a nice walking trail that takes 30 minutes to walk and try it (n=71)  I will make a list of free exercise activities that I want to try (n=40)  I will stand up or go for a walk when I talk on the phone (n=32)  I will find a friend who wants to go for a walk with me (n=29)  I will bike or walk instead of taking the car or bus wherever I go this week (n=19)  I will carry a heavy backpack with my study books on my walk (n=6) | | 635 |
| Alcohol  I will abstain completely from alcohol this week or drink alcohol-free beverages (n=54)  I will drink water every other drink when I consume alcohol this week (n=53)  I will not drink alcohol after midnight – or another specific time that I chose (n=18)  I will abstain from drinking alcohol at home before the pre-party (n=7)  I will not drink more than 4 glasses per evening this week (n=5)  I will only buy half of what I usually buy at Systembolaget (n=5)  I will tell a friend that I aim to drink less and ask them to encourage me (n=5)  I will make a list of activities that one can do instead of drinking alcohol (n=4)  I will apologize to someone I hurt when I was drunk (n=2)  I will calculate how much money alcohol cost me (n=1) | | 154 |
| Smoking  I will, on one occasion, skip cigarettes and drink a glass of water instead (n=10)  I will leave the cigarettes at home when I go out for a short while (n=8)  I will not smoke after breakfast or lunch two days this week (n=7)  I will not smoke before 12.00 PM on one day this week (n=5)  I will tell a friend that I am considering quitting smoking and ask them to encourage me (n=5)  I will smoke less on each cigarette this week (n=5)  I will not smoke my favorite cigarette on two occasions this week (n=3)  I will change cigarette brand and buy a brand that I do not like (n=3)  I will calculate how much money I spend on cigarettes each week (n=2)  I will throw away 3 cigarettes from each pack I buy this week (n=2) | | 50 |
| Total | | **1506** |
