# Supplementary material for: Setting Goals and Accepting Challenges for Behavior Change—Analysis of Participants’ Interactions With a Digital Multiple Health Behavior Intervention: Mixed Methods Study
Source: JMIR Hum Factors. 2025 Aug 29;12:e66208. doi: 10.2196/66208 (PMC12396776; doi:10.2196/66208)
Supplement: Multimedia Appendix 2 [file humanfactors-v12-e66208-s002.docx]

## Setting goals and selecting or self-authoring challenges

Table S1 - Associations between baseline characteristics and setting a goal.

|  | Est. | POA |
| --- | --- | --- |
| Man vs. woman | 0.44 (0.30; 0.66) | > 99.9% |
| Age | 1.01 (0.99; 1.03) | 72.4% |
| Weekly alcohol consumption | 1.03 (0.98; 1.07) | 89.0% |
| Heavy episodic drinking | 0.96 (0.88; 1.04) | 86.3% |
| Average daily fruit and vegetables | 1.04 (0.91; 1.19) | 71.2% |
| Weekly sugary drinks | 1.00 (0.96; 1.03) | 59.8% |
| Weekly candy and snacks | 1.03 (1.00; 1.05) | 98.0% |
| *Body mass index* |  |  |
| Under (<18.5) vs. Normal (18.5-24.9) | 1.27 (0.61; 2.70) | 73.9% |
| Over (25.0-29.9) vs. Normal (18.5-24.9) | 1.03 (0.73; 1.46) | 56.8% |
| Obese (≥30) vs. Normal (18.5-24.9) | 1.16 (0.77; 1.72) | 75.8% |
| Moderate and vigorous physical activity | 0.96 (0.82; 1.11) | 71.4% |
| Weekly cigarettes | 1.12 (0.96; 1.32) | 92.0% |
| Perceived stress | 0.99 (0.94; 1.04) | 66.1% |
| Importance of change | 1.09 (1.00; 1.18) | 97.4% |
| Confidence in ability to change | 1.02 (0.95; 1.10) | 69.4% |
| Knowledge of how to change | 1.04 (0.97; 1.12) | 87.6% |
| **Est.** – Median of the posterior distribution of odds ratios with 95% compatibility intervals.  **POA** – Proportion of the posterior distribution above or below the null in the direction of the median. | | |

Table S2 - Associations between baseline characteristics and selecting or self-authoring a challenge.

|  | Est. | POA |
| --- | --- | --- |
| Man vs. woman | 0.46 (0.30; 0.67) | > 99.9% |
| Age | 1.01 (0.99; 1.03) | 86.2% |
| Weekly alcohol consumption | 1.01 (0.97; 1.05) | 65.5% |
| Heavy episodic drinking | 0.98 (0.90; 1.06) | 67.5% |
| Average daily fruit and vegetables | 1.04 (0.91; 1.20) | 72.1% |
| Weekly sugary drinks | 0.98 (0.95; 1.02) | 82.7% |
| Weekly candy and snacks | 1.02 (0.99; 1.04) | 88.6% |
| *Body mass index* |  |  |
| Under (<18.5) vs. Normal (18.5-24.9) | 1.13 (0.54; 2.36) | 62.7% |
| Over (25.0-29.9) vs. Normal (18.5-24.9) | 0.99 (0.70; 1.39) | 52.0% |
| Obese (≥30) vs. Normal (18.5-24.9) | 1.32 (0.89; 1.97) | 91.4% |
| Moderate and vigorous physical activity | 1.07 (0.92; 1.25) | 81.8% |
| Weekly cigarettes | 1.03 (0.89; 1.19) | 64.3% |
| Perceived stress | 1.03 (0.97; 1.09) | 84.0% |
| Importance of change | 1.07 (0.98; 1.16) | 93.6% |
| Confidence in ability to change | 0.96 (0.89; 1.03) | 87.1% |
| Knowledge of how to change | 1.02 (0.95; 1.10) | 69.4% |
| **Est.** – Median of the posterior distribution of odds ratios with 95% compatibility intervals.  **POA** – Proportion of the posterior distribution above or below the null in the direction of the median. | | |

## Participant characteristics and setting goals and selecting challenges targeting different behaviors

Table S3 – Associations between sex and setting goals and selecting challenges targeting different behaviors

| **Man vs. Woman** | **Est.** | **POA** |
| --- | --- | --- |
| **Set a goal targeting:** | | |
| Alcohol | 0.13 (0.00; 1.38) | 94.9% |
| Diet | 0.29 (0.11; 0.69) | 99.8% |
| PA | 1.51 (0.83; 2.84) | 90.9% |
| Tobacco | 2.93 (0.46; 16.9) | 88.1% |
| Multiple lifestyles | 0.45 (0.11; 1.48) | 89.7% |
| Miscellaneous | 1.53 (0.66; 3.37) | 84.4% |
| **Frequency of ready-made challenges selected and self-authored:** | | |
| Alcohol | 0.55 (0.27; 1.04) | 96.6% |
| Diet | 0.60 (0.41; 0.86) | 99.7% |
| PA | 0.60 (0.39; 0.90) | 99.3% |
| Smoking | 1.31 (0.30; 5.61) | 64.6% |
| Self-authored | 1.72 (0.60; 5.38) | 84.3% |
| **Est.** – Median of the posterior distribution of odds and incidence rate ratios with 95% compatibility intervals.  **POA** – Proportion of the posterior distribution above or below the null in the direction of the median. | | |

Table S4 - Associations between age and setting goals and selecting challenges targeting different behaviors

| Age | Est. | POA |
| --- | --- | --- |
| **Set a goal targeting:** | | |
| Alcohol | 1.13 (1.04; 1.24) | > 99.8% |
| Diet | 1.00 (0.96; 1.03) | 52.0% |
| PA | 0.99 (0.97; 1.02) | 63.0% |
| Tobacco | 0.92 (0.78; 1.04) | 89.7% |
| Multiple lifestyles | 1.00 (0.94; 1.05) | 55.6% |
| Miscellaneous | 0.98 (0.93; 1.02) | 84.7% |
| **Frequency of ready-made challenges selected and self-authored:** | | |
| Alcohol | 1.00 (0.97; 1.04) | 57.0% |
| Diet | 0.99 (0.97; 1.00) | 95.7% |
| PA | 0.99 (0.97; 1.01) | 84.0% |
| Smoking | 0.98 (0.90; 1.08) | 63.6% |
| Self-authored | 1.02 (0.96; 1.08) | 73.8% |
| **Est.** – Median of the posterior distribution of odds and incidence rate ratios with 95% compatibility intervals.  **POA** – Proportion of the posterior distribution above or below the null in the direction of the median. | | |

Table S5 - Associations between weekly alcohol consumption and setting goals and selecting challenges targeting different behaviors

| Weekly alcohol consumption | Est. | POA |
| --- | --- | --- |
| Set a goal targeting: | | |
| Alcohol | 1.07 (0.95; 1.20) | 86.9% |
| Diet | 0.93 (0.86; 1.01) | 95.4% |
| PA | 0.97 (0.92; 1.03) | 82.5% |
| Tobacco | 0.97 (0.75; 1.20) | 62.3% |
| Multiple lifestyles | 1.12 (1.03; 1.22) | 99.5% |
| Miscellaneous | 1.08 (0.99; 1.17) | 96.3% |
| Frequency of ready-made challenges selected and self-authored: | | |
| Alcohol | 1.06 (1.01; 1.12) | 99.5% |
| Diet | 1.00 (0.97; 1.04) | 59.7% |
| PA | 0.96 (0.93; 1.00) | 98.5% |
| Smoking | 1.04 (0.92; 1.18) | 74.3% |
| Self-authored | 1.07 (0.96; 1.20) | 89.6% |
| **Est.** – Median of the posterior distribution of odds and incidence rate ratios with 95% compatibility intervals.  **POA** – Proportion of the posterior distribution above or below the null in the direction of the median. | | |

Table S6 - Associations between heavy episodic drinking and setting goals and selecting challenges targeting different behaviors

| Heavy episodic drinking | Est. | POA |
| --- | --- | --- |
| Set a goal targeting: | | |
| Alcohol | 1.12 (0.91; 1.40) | 86.5% |
| Diet | 0.98 (0.82; 1.14) | 60.2% |
| PA | 1.00 (0.89; 1.12) | 51.2% |
| Tobacco | 0.92 (0.55; 1.35) | 65.1% |
| Multiple lifestyles | 0.91 (0.74; 1.08) | 85.0% |
| Miscellaneous | 0.92 (0.77; 1.06) | 87.7% |
| Frequency of ready-made challenges selected and self-authored: | | |
| Alcohol | 1.11 (1.00; 1.24) | 98.5% |
| Diet | 0.95 (0.88; 1.02) | 93.1% |
| PA | 1.04 (0.97; 1.11) | 87.9% |
| Smoking | 1.09 (0.85; 1.46) | 76.6% |
| Self-authored | 0.79 (0.60; 1.01) | 97.1% |
| **Est.** – Median of the posterior distribution of odds and incidence rate ratios with 95% compatibility intervals.  **POA** – Proportion of the posterior distribution above or below the null in the direction of the median. | | |

Table S7 - Associations between weekly cigarettes and setting goals and selecting challenges targeting different behaviors

| Weekly cigarettes | Est. | POA |
| --- | --- | --- |
| Set a goal targeting: | | |
| Alcohol | 0.58 (0.16; 1.10) | 94.3% |
| Diet | 0.76 (0.49; 1.03) | 95.9% |
| PA | 0.79 (0.62; 0.98) | 98.4% |
| Tobacco | 3.60 (2.35; 6.20) | > 99.9% |
| Multiple lifestyles | 1.05 (0.75; 1.40) | 61.6% |
| Miscellaneous | 0.37 (0.07; 0.91) | 98.9% |
| Frequency of ready-made challenges selected and self-authored: | | |
| Alcohol | 1.03 (0.81; 1.25) | 59.7% |
| Diet | 0.93 (0.82; 1.04) | 90.7% |
| PA | 0.96 (0.84; 1.08) | 76.0% |
| Smoking | 3.32 (2.01; 7.78) | > 99.9% |
| Self-authored | 0.95 (0.66; 1.46) | 61.0% |
| **Est.** – Median of the posterior distribution of odds and incidence rate ratios with 95% compatibility intervals.  **POA** – Proportion of the posterior distribution above or below the null in the direction of the median. | | |

Table S8 - Associations between fruit and vegetables and setting goals and selecting challenges targeting different behaviors

| Average daily fruit and vegetables | Est. | POA |
| --- | --- | --- |
| Set a goal targeting: | | |
| Alcohol | 0.59 (0.27; 1.14) | 93.8% |
| Diet | 0.71 (0.55; 0.90) | 99.8% |
| PA | 1.38 (1.14; 1.67) | > 99.9% |
| Tobacco | 0.57 (0.21; 1.21) | 92.1% |
| Multiple lifestyles | 0.84 (0.58; 1.15) | 85.4% |
| Miscellaneous | 0.95 (0.69; 1.27) | 62.7% |
| Frequency of ready-made challenges selected and self-authored: | | |
| Alcohol | 1.03 (0.85; 1.24) | 62.7% |
| Diet | 0.88 (0.79; 0.97) | 99.5% |
| PA | 1.16 (1.05; 1.28) | 99.8% |
| Smoking | 1.64 (1.00; 2.87) | 97.7% |
| Self-authored | 0.88 (0.60; 1.31) | 73.8% |
| **Est.** – Median of the posterior distribution of odds and incidence rate ratios with 95% compatibility intervals.  **POA** – Proportion of the posterior distribution above or below the null in the direction of the median. | | |

Table S9 - Associations between weekly sugary drinks and setting goals and selecting challenges targeting different behaviors

| Weekly sugary drinks | Est. | POA |
| --- | --- | --- |
| Set a goal targeting: | | |
| Alcohol | 0.96 (0.73; 1.16) | 65.2% |
| Diet | 0.98 (0.93; 1.04) | 73.7% |
| PA | 0.99 (0.94; 1.04) | 69.3% |
| Tobacco | 1.07 (0.91; 1.24) | 80.2% |
| Multiple lifestyles | 1.09 (1.00; 1.19) | 97.7% |
| Miscellaneous | 0.94 (0.85; 1.02) | 91.7% |
| Frequency of ready-made challenges selected and self-authored: | | |
| Alcohol | 0.96 (0.89; 1.03) | 86.3% |
| Diet | 0.99 (0.97; 1.02) | 64.2% |
| PA | 0.99 (0.97; 1.02) | 64.8% |
| Smoking | 1.04 (0.89; 1.21) | 69.8% |
| Self-authored | 1.05 (0.96; 1.16) | 86.1% |
| **Est.** – Median of the posterior distribution of odds and incidence rate ratios with 95% compatibility intervals.  **POA** – Proportion of the posterior distribution above or below the null in the direction of the median. | | |

Table S10 - Associations between weekly candy and snacks and setting goals and selecting challenges targeting different behaviors

| Weekly candy and snacks | Est. | POA |
| --- | --- | --- |
| Set a goal targeting: | | |
| Alcohol | 0.97 (0.87; 1.07) | 70.8% |
| Diet | 1.00 (0.97; 1.04) | 57.4% |
| PA | 1.00 (0.98; 1.04) | 71.0% |
| Tobacco | 0.99 (0.89; 1.10) | 54.3% |
| Multiple lifestyles | 0.99 (0.93; 1.04) | 67.3% |
| Miscellaneous | 0.97 (0.92; 1.02) | 87.2% |
| Frequency of ready-made challenges selected and self-authored: | | |
| Alcohol | 1.00 (0.97; 1.03) | 50.6% |
| Diet | 1.02 (1.00; 1.04) | 99.4% |
| PA | 0.99 (0.98; 1.02) | 56.5% |
| Smoking | 1.03 (0.95; 1.13) | 77.5% |
| Self-authored | 1.04 (0.98; 1.10) | 91.4% |
| **Est.** – Median of the posterior distribution of odds and incidence rate ratios with 95% compatibility intervals.  **POA** – Proportion of the posterior distribution above or below the null in the direction of the median. | | |

Table S11 - Associations between body mass index and setting goals and selecting challenges targeting different behaviors

| Body mass index | Est. | POA |
| --- | --- | --- |
| Set a goal targeting: | | |
| *Alcohol:* |  |  |
| Under (<18.5) vs. Normal (18.5-24.9) | 0.20 (0.0; 3.9) | 83.3% |
| Over (25.0-29.9) vs. Normal (18.5-24.9) | 0.27 (0.06; 1.00) | 97.6% |
| Obese (≥30) vs. Normal (18.5-24.9) | 0.02 (0.0; 0.30) | 99.9% |
| *Diet:* |  |  |
| Under (<18.5) vs. Normal (18.5-24.9) | 2.36 (0.76; 7.16) | 93.3% |
| Over (25.0-29.9) vs. Normal (18.5-24.9) | 1.08 (0.61; 1.90) | 60.6% |
| Obese (≥30) vs. Normal (18.5-24.9) | 1.80 (0.98; 3.33) | 97.2% |
| *PA:* |  |  |
| Under (<18.5) vs. Normal (18.5-24.9) | 0.76 (0.27; 2.04) | 70.7% |
| Over (25.0-29.9) vs. Normal (18.5-24.9) | 1.22 (0.77; 1.93) | 80.2% |
| Obese (≥30) vs. Normal (18.5-24.9) | 1.26 (0.74; 2.14) | 79.9% |
| *Tobacco:* |  |  |
| Under (<18.5) vs. Normal (18.5-24.9) | 0.01 (0.0; 0.67) | 98.6% |
| Over (25.0-29.9) vs. Normal (18.5-24.9) | 1.13 (0.19; 5.80) | 56.3% |
| Obese (≥30) vs. Normal (18.5-24.9) | 0.54 (0.06; 4.13) | 71.8% |
| *Multiple lifestyles:* |  |  |
| Under (<18.5) vs. Normal (18.5-24.9) | 0.69 (0.10; 2.87) | 67.9% |
| Over (25.0-29.9) vs. Normal (18.5-24.9) | 0.92 (0.42; 1.95) | 58.4% |
| Obese (≥30) vs. Normal (18.5-24.9) | 0.16 (0.03; 0.61) | 99.8% |
| *Miscellaneous:* |  |  |
| Under (<18.5) vs. Normal (18.5-24.9) | 1.15 (0.24; 4.24) | 57.6% |
| Over (25.0-29.9) vs. Normal (18.5-24.9) | 0.92 (0.42; 1.92) | 58.6% |
| Obese (≥30) vs. Normal (18.5-24.9) | 1.02 (0.4; 2.44) | 51.3% |
| Frequency of ready-made challenges selected and self-authored: | | |
| *Alcohol:* |  |  |
| Under (<18.5) vs. Normal (18.5-24.9) | 0.71 (0.25; 1.79) | 76.6% |
| Over (25.0-29.9) vs. Normal (18.5-24.9) | 0.85 (0.52; 1.37) | 74.1% |
| Obese (≥30) vs. Normal (18.5-24.9) | 0.60 (0.32; 1.09) | 95.5% |
| *Diet:* |  |  |
| Under (<18.5) vs. Normal (18.5-24.9) | 0.55 (0.30; 0.98) | 98.0% |
| Over (25.0-29.9) vs. Normal (18.5-24.9) | 1.08 (0.85; 1.38) | 72.9% |
| Obese (≥30) vs. Normal (18.5-24.9) | 0.92 (0.70; 1.22) | 72.3% |
| *PA:* |  |  |
| Under (<18.5) vs. Normal (18.5-24.9) | 0.54 (0.28; 1.02) | 97.2% |
| Over (25.0-29.9) vs. Normal (18.5-24.9) | 0.82 (0.63; 1.08) | 91.3% |
| Obese (≥30) vs. Normal (18.5-24.9) | 0.75 (0.56; 1.02) | 96.6% |
| *Smoking:* |  |  |
| Under (<18.5) vs. Normal (18.5-24.9) | 0.05 (0.0; 1.48) | 95.5% |
| Over (25.0-29.9) vs. Normal (18.5-24.9) | 1.38 (0.44; 4.72) | 71.3% |
| Obese (≥30) vs. Normal (18.5-24.9) | 0.06 (0.00; 0.60) | 99.4% |
| *Self-authored:* |  |  |
| Under (<18.5) vs. Normal (18.5-24.9) | 0.88 (0.18; 4.99) | 56.2% |
| Over (25.0-29.9) vs. Normal (18.5-24.9) | 0.83 (0.36; 1.95) | 66.6% |
| Obese (≥30) vs. Normal (18.5-24.9) | 1.55 (0.62; 3.95) | 82.4% |
| **Est.** – Median of the posterior distribution of odds and incidence rate ratios with 95% compatibility intervals.  **POA** – Proportion of the posterior distribution above or below the null in the direction of the median. | | |

Table S12 - Associations between moderate and vigorous physical activity and setting goals and selecting challenges targeting different behaviors

| Moderate and vigorous physical activity | Est. | POA |
| --- | --- | --- |
| Set a goal targeting: | | |
| Alcohol | 1.25 (0.68; 2.15) | 77.6% |
| Diet | 1.72 (1.35; 2.19) | > 99.9% |
| PA | 0.55 (0.43; 0.69) | > 99.9% |
| Tobacco | 1.32 (0.62; 2.59) | 77.7% |
| Multiple lifestyles | 0.88 (0.58; 1.27) | 73.9% |
| Miscellaneous | 1.34 (0.99; 1.78) | 97.2% |
| Frequency of ready-made challenges selected and self-authored: | | |
| Alcohol | 1.07 (0.86; 1.31) | 72.2% |
| Diet | 1.08 (0.97; 1.20) | 92.0% |
| PA | 0.77 (0.67; 0.88) | > 99.9% |
| Smoking | 1.07 (0.61; 1.86) | 59.9% |
| Self-authored | 1.42 (0.96; 2.17) | 95.8% |
| **Est.** – Median of the posterior distribution of odds and incidence rate ratios with 95% compatibility intervals.  **POA** – Proportion of the posterior distribution above or below the null in the direction of the median. | | |

Table S13 - Associations between perceived stress and setting goals and selecting challenges targeting different behaviors

| Perceived stress | Est. | POA |
| --- | --- | --- |
| Set a goal targeting: | | |
| Alcohol | 0.83 (0.67; 1.02) | 96.3% |
| Diet | 0.98 (0.90; 1.06) | 71.2% |
| PA | 1.02 (0.95; 1.09) | 70.0% |
| Tobacco | 0.81 (0.60; 1.07) | 93.4% |
| Multiple lifestyles | 0.91 (0.80; 1.03) | 93.1% |
| Miscellaneous | 1.21 (1.09; 1.36) | > 99.9% |
| Frequency of ready-made challenges selected and self-authored: | | |
| Alcohol | 0.95 (0.88; 1.02) | 92.0% |
| Diet | 0.96 (0.93; 1.00) | 97.3% |
| PA | 0.98 (0.94; 1.02) | 85.7% |
| Smoking | 1.30 (1.04; 1.70) | 99.0% |
| Self-authored | 1.09 (0.95; 1.28) | 90.5% |
| **Est.** – Median of the posterior distribution of odds and incidence rate ratios with 95% compatibility intervals.  **POA** – Proportion of the posterior distribution above or below the null in the direction of the median. | | |

Table S14 - Associations between importance of change and setting goals and selecting challenges targeting different behaviors

| Importance of change | Est. | POA |
| --- | --- | --- |
| Set a goal targeting: | | |
| Alcohol | 1.43 (0.99; 2.21) | 97.0% |
| Diet | 1.03 (0.97; 1.10) | 85.9% |
| PA | 0.94 (0.84; 1.06) | 84.2% |
| Tobacco | 0.95 (0.62; 1.58) | 59.4% |
| Multiple lifestyles | 1.04 (0.85; 1.29) | 63.4% |
| Miscellaneous | 0.93 (0.78; 1.11) | 78.7% |
| Frequency of ready-made challenges selected and self-authored: | | |
| Alcohol | 0.93 (0.83; 1.05) | 87.5% |
| Diet | 1.03 (0.97; 1.09) | 81.3% |
| PA | 0.97 (0.91; 1.04) | 79.0% |
| Smoking | 0.82 (0.58; 1.14) | 88.7% |
| Self-authored | 0.79 (0.62; 0.98) | 98.4% |
| **Est.** – Median of the posterior distribution of odds and incidence rate ratios with 95% compatibility intervals.  **POA** – Proportion of the posterior distribution above or below the null in the direction of the median. | | |

Table S15 - Associations between confidence in ability to change and setting goals and selecting challenges targeting different behaviors

| Confidence in ability to change | Est. | POA |
| --- | --- | --- |
| Set a goal targeting: | | |
| Alcohol | 1.03 (0.79; 1.38) | 58.3% |
| Diet | 1.03 (0.92; 1.15) | 68.0% |
| PA | 0.98 (0.90; 1.08) | 65.6% |
| Tobacco | 0.89 (0.61; 1.27) | 73.2% |
| Multiple lifestyles | 1.03 (0.88; 1.23) | 64.6% |
| Miscellaneous | 1.03 (0.89; 1.19) | 64.0% |
| Frequency of ready-made challenges selected and self-authored: | | |
| Alcohol | 0.98 (0.88; 1.08) | 67.5% |
| Diet | 1.00 (0.95; 1.05) | 53.4% |
| PA | 1.04 (0.98; 1.10) | 89.2% |
| Smoking | 1.07 (0.79; 1.49) | 66.7% |
| Self-authored | 0.94 (0.79; 1.11) | 77.8% |
| **Est.** – Median of the posterior distribution of odds and incidence rate ratios with 95% compatibility intervals.  **POA** – Proportion of the posterior distribution above or below the null in the direction of the median. | | |

Table S16 - Associations between knowledge of how to change and setting goals and selecting challenges targeting different behaviors

| Knowledge of how to change | Est. | POA |
| --- | --- | --- |
| Set a goal targeting: | | |
| Alcohol | 0.72 (0.52; 0.98) | 98.1% |
| Diet | 1.04 (0.92; 1.18) | 75.3% |
| PA | 0.94 (0.85; 1.04) | 89.0% |
| Tobacco | 0.76 (0.49; 1.14) | 91.0% |
| Multiple lifestyles | 1.11 (0.93; 1.33) | 87.5% |
| Miscellaneous | 1.05 (0.89; 1.23) | 71.0% |
| Frequency of ready-made challenges selected and self-authored: | | |
| Alcohol | 0.95 (0.86; 1.05) | 83.9% |
| Diet | 1.02 (0.97; 1.07) | 75.3% |
| PA | 0.99 (0.93; 1.05) | 66.4% |
| Smoking | 1.09 (0.82; 1.44) | 72.4% |
| Self-authored | 1.14 (0.95; 1.39) | 91.6% |
| **Est.** – Median of the posterior distribution of odds and incidence rate ratios with 95% compatibility intervals.  **POA** – Proportion of the posterior distribution above or below the null in the direction of the median. | | |
