# Supplementary material for: Setting Goals and Accepting Challenges for Behavior Change—Analysis of Participants’ Interactions With a Digital Multiple Health Behavior Intervention: Mixed Methods Study
Source: JMIR Hum Factors. 2025 Aug 29;12:e66208. doi: 10.2196/66208 (PMC12396776; doi:10.2196/66208)
Supplement: Multimedia Appendix 3 [file humanfactors-v12-e66208-s003.docx]

| Summary of health behavior goals (n=607) | | | | |
| --- | --- | --- | --- | --- |
| Physical activity (n=302)  Minutes and type of activity per day (n=143)  Number of training days or activities per week (n=108) | | | Minutes or hours of training per week (n=23)  Be more physically active (n=23)  Follow the plan (n=5) | |
| Dietary behavior (n=140)  Avoid sweets, sugar, and snacks (n=50)  Eat healthier (n=40) | | | Eat more fruit and vegetables (n=38)  Drink less sugar (n=12) | |
| Miscellaneous behavior (n=72)  Performance goals (n=26)  Mental health goals (n=18) | | | Sleep behavior goals (n=14)  Physical body goals (n=10)  Mobile phone behavior goals (n=10) | |
| Multiple health behavior (n=53)  Diet & Physical activity goals (n=22)  Health behaviors in combinations of three or more (n=10)  Physical activity & Alcohol goals (n=9) | | | Physical activity & Smoking goals (n=6)  Physical activity & Other routine goals (n=3)  Alcohol, Smoking, Diet goals (n=3) | |
| Alcohol consumption (n=19)  Amount limits: 'Not more than', 'maximum units', or 'less than' (n=14) | | | Restricted drinking: To not drink at all, or during a specific time frame (n=5)  Unspecified limit: Just less (n=1) | |
| Tobacco behavior (n=17)  Smoking goals: Defined amount and time limits (n=12) | | | Smoking goals: To cut down, to quit or to not smoke at all (n=3)  Snuff goals: Consumption limits and reducing nicotine strength (n=2) | |
| Summary of strategies to achieve behavior change (n=607) | | | | |
| Physical activity (n=302)   - Planning and preparations. - Specified and time-framed physical activity. - Specified type of physical activity. - Not specified what, when or how. | **Dietary behavior** (n=140)   - Restrictions and Replacements. - Planning and preparations. - Availability. - Add fruit and vegetables to meals. - Distractions. | **Multiple health** (n=53)   - Restrictions. - Routines. - Preparations. - Prioritization. - Recovery.   **Alcohol consumption** (n=19)   - Replacements. - Adjust drinking habits. - Availability. | | **Tobacco behavior** (n=17)   - Restrictions and awareness. - Substitutes. - Unspecified.   **Miscellaneous behavior** (n=72)   - Sleep routines. - Self-reflection. - Physical activity. - Task-orientation. - Mobile phone discipline. - Making plans. |
| Summary of motivational self-talk to encourage behavior change (n=607) | | | | |
| Physical activity (n=302)   - Post-activity benefits. - Self-encouragement. - Long-term value. - Effort vs. reward. - Just do it. - Personal satisfaction and enjoyment. - Goal-orientation. - Past success. - Unspecified. | **Dietary behavior** (n=140)   - Health benefits. - Self-encouragement. - Better alternatives. - Resist cravings. - Goal-orientation. - I don´t know. | **Multiple health** (n=53)   - Health benefits. - Self-persuasion and encouragement. - Goal-orientation.   **Alcohol consumption** (n=19)   - Well-being and health benefits. - Self-control. - Unspecified. | | **Tobacco behavior** (n=17)   - Health benefits. - Self-encouragement. - Unspecified.   **Miscellaneous behavior** (n=72)   - Health benefits and sustainability. - Self-encouragement. - Time-management. - Self-value. - I don´t know. |
| Summary of rewards to treat oneself with after accomplishing behavior goals (n=607) | | | | |
| Physical activity (n=302)   - Indulgence. - Shopping. - Self-appreciation. - Relaxation. - Self-care. - Activities. - Entertainment. - Socializing. - Nothing or I don´t know. - Economy. | **Dietary behavior** (n=140)   - Indulgence. - Shopping. - Relaxation. - Self-satisfaction. - Activities. - Economy. - Socializing. - Nothing or I don´t know. - A treat for me. | **Multiple health** (n=53)   - Shopping. - Indulgence. - Relaxation. - Self-satisfaction. - Enjoyment. - I don´t know.   **Alcohol consumption** (n=19)   - Shopping. - Feelgood. - Enjoyments. | | **Tobacco behavior** (n=17)   - Shopping. - Enjoyment and self-satisfaction. - Smoking. - Indulgence. - I don´t know.   **Miscellaneous behavior** (n=72)   - Shopping. - Self-satisfaction. - Indulgence. - Relaxation. - Socializing. - Nothing. - Economy. - Travel. |

| Physical activity goals (n=302) | In the upcoming week, my goal is to… |
| --- | --- |
| *Minutes and type of activity, per day:* (N=143)  > 20 minutes (n=11)  30 minutes (n=48)  40-45 minutes (n=7)  60 minutes (n=28)  Specified activities per day (getting out of the house, some kind of movement, walking or steps/day, jogging, exercising, gym training, cycling, rowing, push-ups, stretching, online workouts (n=49) | *”…walk at least 20 minutes per day”*  *”…raise my pulse 30 min/day”*  *”…engage in moderately intense movement for at least 40 min/day”*  *”…spend 1 hour outdoors per day”*  *”go for a walk every morning before studying”*  *”…cycling to work every day”*  *”…aim for at least 10 000 steps per day”* |
| *Number of training days or activities, per week:* (N=108)  One time (n=18)  Two times (n=30)  Three times (n=41)  Four times (n=11)  Five times or more (n=8)  Activities students aim to engage in: walking, powerwalking, running, swimming, cycling, dancing, gym/resistance training, vigorous activity training, being active, movement, exercising. | *”…walk to the university once this week”*  *”…to conduct two gym sessions”*  *”…do vigorous physical activity 3 days/week”*  *”…exercise at least 4 times this week”*  *”work out at the gym 2 times, and take 5 daily walks”* |
| *Minutes or hours of training per week:*  Defined in minutes, hours or distance (n=23) | *”…be physically active for 150 minutes this week”*  *”…walk at least 2500 steps”* |
| *To be more physically active:*  Involves a general focus on ‘being active’ by increasing physical activity, or movement, by starting or resuming training, walks, stair-walking, cycling, running, or going to the gym (n=23) | *”…to work out more”*  *”…to get outside, preferably for a walk”*  *”…go for a run, even if it feels tough”* |
| *To follow the plan:*  Involves a focus on consistency, completion and adherence to training schedules. Students aim to maintain regular practice, complete rehabilitation exercises, or continue to use a training app (n=5) | *”…follow my training schedule”*  *”…to be consistent with training”* |
| Strategies to achieve physical activity behavior change (n=302) | **In the upcoming week, I will undertake these two actions**  **to progress towards my goals…** |
| *Planning and preparations:* Involves ‘planning to make plans’ which includes scheduling physical activity in advance and preparing such as structuring their study schedule, preparing training clothes, adjusting sleep and meal routines, and planning to use reminders and motivational tools such as podcasts while training. It also involves planning to prioritize these activities and seeking social support to stay motivated (n=131) | *”…get an overview of the week´s planning and plan in advance when to go out”*  *”…book sessions at the gym plan training together with a friend”*  *”…by planning when I will work out and trying to get my sleep in order”*  *”…by booking training sessions in my calendar and ask [Name] to remind me”*  *”…by working out with a friend and preparing my bag with workout clothes”*  *”…by sleeping on time and making a lunchbox the day before”*  *”…by setting reminders and finding something good to listen to”* |
| *Specified and time-framed physical activity:* Involves defining the physical activity to be undertaken, its timing and execution (*what*, *when* and *how* to do it). For example walking or exercising in the morning, during study breaks, or after work/school. Or planning to use active transportation, such as walking or cycling, instead of taking the bus. It also involves preparing for and prioritizing these activities (n=113) | *”…take a 15-minute walk in the morning and one after I have studied”*  *” take a walk every day between study sessions and exercise in the morning”*  *”…get off the train 2 stations early and eat 3 fruits”*  *”…go out regardless of the weather and walk home after school”*  *”…walk in the morning and avoid buying soda from Monday to Thursday”* |
| *Specified type of physical activity*: Involves defining the kind of physical activity intended to be executed (*what* to be done). For example walking, either alone or in combination with other activities like going to the gym, strength exercises, running, yoga or cycling (n=50) | *”…by walking and going to the gym”*  *”…by taking short walks and being more physically active”*  *”…by going out for a run and doing simple strength exercises”* |
| Not specified what, when or how (n=8) | *”…by exercising and eating well”* |
| Motivational self-talk to encourage physical activity behavior change (n=302) | **This is how I will remind and encourage myself when motivation wavers.**  **If it feels tough, I will tell myself that…** |
| *Post-activity benefits:* Reminding oneself of the value and benefits previously experienced from physical activity, such as positive emotions, health improvements, enhanced focus, increased energy, and overall well-being (n=112) | *”I always feel better after exercising”*  *”…It feels so good afterwards, and it will help me focus when I´m studying”*  *”…my body needs it to cope”*  *”…when you come back home again you are alert and energized all evening”* |
| *Self-encouragement:* Involves motivating oneself with positive affirmations like ’you can do it’. It also involves pushing oneself to work hard, overcoming barriers, taking personal responsibility, enduring tough feelings, and believing that hard work pays off (n=50) | *”…you CAN DO IT! I BELIEVE IN YOU!”*  *”…I can do it, I want to do it, I will do it”*  *”… aiming to be a superhero - only you can make the change!”*  *”…the obstacle is the way”* |
| *Long-term value:* Involves focusing on future benefits, health improvements, and personal well-being. It also involves self-compassion in terms of being worthy of feeling good, and self-pride that comes from being physically active (n=34) | *”…think long-term about your health, it´s worth it”*  *”…I am strong and great and deserve to feel good”*  *”… you are doing it for yourself, don´t prioritize other things before yourself”*  *”…I will be so proud of myself afterwards”* |
| *Effort vs. reward:* Involves rationalizing and reminding oneself that the task should be viewed as manageable, won´t require much effort, and won´t be overly time-consuming, and encouraging oneself that even small efforts are valuable (n=28) | *”…it is not so hard”*  *”…it is just 15 minutes. I can sacrifice that even if it´s boring”*  *”…a little goes a long way”*  *”…a small step is better than nothing, cycling is more fun than idly watching tv”* |
| *Just do it:* Involves pushing oneself to get a grip and take action without overthinking, encouraging personal responsibility and sternness (n=24) | *”…get a grip, it goes quickly and it´s fun!”*  *”…just do it, pretend like life depends on it!”* |
| *Personal satisfaction and enjoyment:* Involves reminding oneself that physical activity is done for personal satisfaction and because one wants to, not because one has to, and highlights previously experienced joy of being active (n=17) | *”…remind myself that I do this for my own sake”*  *”…I do this because I want to, it´s the long-term benefits that I´m after”*  *”…it will be fun when you get there”*  *”…I usually enjoy training. I want to get in shape and become stronger”* |
| *Goal-orientation:* Reminding oneself to focus on the goal, whether it is to succeed with a task such as studies, or to improve one’s physical appearance, lose weight or become stronger (n=16) | *”…this will make me pass my exam”*  *”…you want a healthier heart and a better-looking body”*  *”…imagine how strong I will become! Think of the dumbbells”* |
| *Past success:* Reminding oneself of previous accomplishments in being physically active and associated positive feelings (n=13) | *” …I managed to exercise last week and it felt so much better”*  *”…I have done it before, I will succeed this time too”* |
| Unspecified (n=8) | *”…it is time to take some fresh air”* |
| Rewards to treat oneself with after accomplishing physical activity behavior goals (n=302) | **When I have achieved my goal, I will reward myself with…** |
| *Indulgence:* Something good to eat, such as a delicious dinner, something special like sushi, chanterelles, exotic fruit, or something nice to drink such as barista coffee, energy drink, or snacks (n=70) | ”…*when I have walked every day all week, I will arrange a nice dinner”*  *”…a luxury coffee on a study day”*  *”…to buy kombucha and a bar of chocolate”* |
| *Shopping:* Buy oneself a reward such as training clothes, -shoes, or -products, or clothes, bags, flowers, beauty products, interior decoration, or crafts, beads, pens and acrylic paint (n=62) | *”…something that proves that I did good”*  *”…treat myself to new training clothes”*  *”…when I have been active for 60 min every day this week, I will buy tulips”* |
| *Self-appreciation:* Involves self-recognition and proud emotions towards oneself such as a pat on the back, complimenting oneself and celebrating after achieving a goal (=55) | *”…a smile and a high five in the mirror”*  *”…then I should just be satisfied with having succeeded”*  *”…confirmation that I can”* |
| *Relaxation:* Indulge in relaxing and recovering activities such as taking a day off, through sleep, rest, and leisure activities (n=28) | *”… a calm weekend with a sleep-in”*  *”…a day where I can relax and do what I want”* |
| *Self-care:* Involves self-care activities such as taking a bath, shower, applying a face mask, foot bath, massage or spa-treatments (n=24) | *”…when I have achieved my goal, I will make a massage reservation”*  *”…a study-free evening and a long, warm shower”* |
| *Activities:* Leisure activities one enjoys such as going to the forest, visiting the rosary, crafting, fixing things at home, listening to music, and travelling (n=17) | *”…to go swim on Friday morning”*  *”…give myself time to paint and draw”*  *”…time to listen to music and just be happy”* |
| *Entertainment:* Involves rewarding oneself with entertainment activities such as watching a favorite series, enjoying a movie night, visiting the cinema, watching a musical, or time on the computer (n=16) | *”…then I will treat myself to watch a series”*  *”…watch a movie and eat something good”*  *”…unlimited time on the computer”* |
| *Socializing:* Involves spending quality time with loved ones and enjoying shared activities (n=13) | *”…get a big hug from [Name] and [Name] and do something fun together”*  *”…just fully enjoy the holiday season with my family”* |
| Nothing or I don´t know (n=12) | *”…I don´t want to give myself anything”* |
| *Economy:* Involves rewarding oneself with a treat or saving up for something larger (n=5) | *”…buy a pair of pants on Thursday with saved bus money”*  *”…invest some money or buy something”* |

| **Dietary behavior goals (n=140)** | **In the upcoming week, my goal is to…** |
| --- | --- |
| ***Avoid sweets, sugar, and snacks:*** Involves avoiding, reducing, limiting, or trying to replace the consumption, or purchase of candy, snacks, and other sugary and fatty products. For instance, setting a maximum number of times these items are allowed to be consumed, or designating specific days (such as weekends) for consumption (n=50) | *”not eat snacks or sweets on weekdays”*  *”eat less unhealthy stuff”*  *”only eat cookies/sweets for a maximum of 3 times”*  *”not eat sugar and swap to fruit and berries instead”* |
| ***Eat healthier:*** Involves planning ‘to eat healthier’ which is expressed in that sense alone or in terms of modified 'eating behavior'. For example aiming to eat more nutritious, eating more regularly throughout the day, planning and preparing cooked meals, eating breakfast, making lunchboxes, avoiding take-away, or adjusting portion sizes (n=40) | *”…eat breakfast every morning”*  *”…eat two cooked meals a day”*  *”…eat healthy [bra] at least five out of seven days”*  *”…eat a moderate amount and nutritiously”*  *”…to drink more water and not order take-away junk food”* |
| ***Eat more fruit and vegetables:*** Involves aiming to consume a certain number of fruits and/or vegetables daily, to incorporate fruits and vegetables into every meal, and to replace dessert with fruits. Or to increase weekly intake without specifying when, or how much (n=38) | *””…eat a fruit every day”*  *”…buy and eat more fruit”*  *”…eat 100 grams of fruit and vegetables every day”*  *”…eat fruit and vegetables with every meal”* |
| ***Drink less sugar:*** Involves avoiding or drinking less soda, sweetened beverages, and carbonated drinks, and switch to drinking water (n=12) | *”…drink less soda and more water”* |
| **Strategies to achieve dietary behavior change (n=140)** | **In the upcoming week, I will undertake these two actions**  **to progress towards my goals…** |
| ***Restrictions and Replacements:*** Refrain from buying unhealthy choices or eating certain foods, substituting them with healthier options like fruit, vegetables, water, and tea when having cravings. Keeping track of food by registration and being physically active (n=45) | *”…not buy it on weekdays and make sure that I have other things to snack on”*  *”not buy buns and ice-cream and choose fruit, berries and vegetables instead”*  *”not buy sweets/cookies and walk my 15 min-round x 3 times”*  *”…register everything before I eat and not buy anything when out”* |
| ***Planning and preparations:*** Involves preparing such as planning, cooking, and eating healthy food regularly. Includes swapping to healthier choices, adding more fruit and vegetables, making food plans and preparation of meals and lunchboxes, setting reminders to eat regularly, and being physically active (n=37) | *”…add fruit and/or vegetables to every meal and eat regularly – less snacking”*  *”…buy fruits to eat instead and eat proper breakfast, lunch and dinner”*  *”…make my own meals and plan in advance what I shall cook”*  *”go for a run at least once and make a lunchbox”*  *”…wake up on time and eat breakfast straight ahead”* |
| ***Availability:*** Increasing the availability of healthy alternatives such as fruits and vegetables by purchasing them to have at home, bringing them to the university, planning on how to incorporate vegetables into meal, and always having a water bottle at hand (n=34) | *”…buy fruit and bring with me to university”*  *”…write ’eat fruit’ on my to do list and make sure there are fruit available at home”*  *”…buy fruit and buy less sweets”*  *”…plan vegetables to every meal and to buy vegetables that I like”* |
| ***Add fruit and vegetables to meals:*** Involves incorporating more fruits and vegetables into meals such as for breakfast and as a snack, increasing water intake and enhancing physical activity (n=16) | *”…drink smoothies for breakfast and eat fruit as a snack”*  *”eat more fruit and engage in movement when I get tired”*  *”…drink plenty of water and eat more fruit”* |
| ***Distraction:*** Involves staying distracted with other activities as a substitute for snacking (n=8) | *”…leisure time, walking, listening to music, events, hanging out with others, taking breaks, talking to my kids”* |
| **Motivational self-talk to encourage dietary behavior change (n=140)** | **This is how I will remind and encourage myself when motivation wavers.**  **If it feels tough, I will tell myself that…** |
| ***Health benefits:*** Reminding themselves that healthier choices lead to long-term benefits, including improved physical and mental well-being, energy levels, mood, focus, digestion, physical appearance, reduced blood pressure, and cost savings. Encouraging persistence despite the initial struggle and discouraging stress eating, junk food and excessive sugar consumption due to health setbacks (n=68) | *”I will be feeling so much better if I don´t snack and avoid sweets”*  *”…it is good for me! And for my wallet”*  *”…it is better for my gut and it feels better in my body”*  *”…your body and mental health will be feeling much better from more greens”*  *”…takeaway is bad for my health and looks, that´s why I will cook”*  *”…you will be feeling better in your gut and your head if you manage this”* |
| ***Self-encouragement:*** Involves uplifting oneself with affirmations like ’you can do it’, reminding oneself that only a small effort is required, that one has accomplished before, and encouraging perseverance to foster a sense of pride towards oneself in the long-term (n=21) | *”…believe in yourself, I believe in you, why aren´t you doing it to?”*  *”…think of how satisfied I will be with myself afterwards”*  *”…every small step are steps on the way to success”*  *”…I have the energy to prepare vegetables, it doesn´t take long”* |
| ***Better alternatives:*** Involves reminding oneself that there are better food options such as fruit, vegetables, drinking water and that they prefer real, home cooked food (n=18) | *”…I actually like cooked food better than sweets and feel better afterwards”*  *”…I actually prefer and feel better with good, cooked food”*  *”…I have so much more energy when I´ve had breakfast”* |
| ***Resist cravings:*** Involves employing strategies to combat craving, such as setting a timer, eating a fruit, or distracting oneself. Reminding and encouraging oneself that succumbing to the craving will not contribute to better well-being (n=18) | *”…I just need to get through this, it´s worth it”*  *”…It´s going to feel good tonight if I abstain now”*  *”…It´s not worth it! I won´t be happier tomorrow, rather anxiety!”*  *”…that I don´t need sweets to feel good”* |
| ***Goal-orientation:*** Involves reminding oneself to stay focused on the long-term benefits to their body and health, and thinking of the reasons behind making this change (n=12) | *”…think long-term! Think about your body and health!*  *”…I should think about what my goal is and why I´m doing what I´m doing”*  *”…that your body will live for many more years”* |
| I don´t know (n=3) | *I don´t know* |
| **Rewards to treat oneself with after accomplishing dietary goal (n=140)** | **When I have achieved my goal, I will reward myself with…** |
| ***Indulgence:*** Something good to eat, such as cooking one´s favorite dinner, baking, or eating out, buying fruit and berries, or looking forward to treating oneself with an unhealthy snack at the weekend (n=43) | *”…buy fruit that I crave even if it´s a bit more expensive”*  *”…treat myself to eating out with my partner”*  *”…indulge myself on the weekend”* |
| ***Shopping:*** Buy something for oneself such as clothing, earrings, a bag, earphones, water bottle, book, flower, or interior decoration (n=35) | *”…I will set aside 300 SEK for shoes for myself”*  *”…I am going to buy a new pair of stylish trousers”* |
| ***Relaxation:*** Indulge in relaxing activities such as taking a bath, shower, massage, alone-time, time for reflection, meditation, or watching favorite series, movies, or playing computer games (n=20) | *”…a quit night with one of my favorite movies”*  *”…some time for reflection”*  *”…at home spa”* |
| ***Self-satisfaction:*** Involves positive emotions such as feeling happy, satisfied, proud, joy, and satisfaction, praise oneself, a mental boost or hug, and a pat on the shoulder (n=18) | *”…be pride of myself”*  *”…feeling joy and satisfaction in succeeding”*  *”…enjoying life with the new habit”* |
| ***Activity:*** Physical activities one enjoys such as going to the gym, paddleboarding, spending time in nature, walking or dancing (n=6) | *”…a well-deserved walk in the forest”*  *”…some self-appreciation and put on music and dance”* |
| ***Economy:*** Invest or save money not spent on soda or take-away (n=5) | *”…save the money I didn´t spend on drinks”* |
| ***Socializing:*** Spending enjoyable time hanging out with friends (n=5) | *”…treat myself with the pleasure of spending time with my friends”* |
| Nothing or I don´t know (n=5) | *”…nothing, this means that I can manage the following week as well”* |
| ***A treat for me:*** Involves booking an appointment with a hairdresser, a tattoo artist, or buying a piercing (n=3) | *”…treat myself with an appointment at the hairdresser and have my hair dyed”* |

| **Miscellaneous behavior goals (n=72)** | **In the upcoming week, my goal is to…** |
| --- | --- |
| ***Performance goals:***  Enhance studying, fulfil commitments and manage finances, by being present at university, attending lectures, planning and shaping routines such as setting a fixed time for studying, and by prioritizing and completing tasks (n=26) | *”… study at least 4 hours a day”*  *”…attend every lecture”*  *”…not postpone study tasks and to not get hung up on my phone”*  *”…create a morning routine”*  *”…prioritize my tasks every day”* |
| ***Mental health goals:***  Self-care and mindset goals with the aim to improve personal care, to focus on well-being, life-balance, meditation and journalling (n=18) | *”…take good care of myself”*  *“…shall focus more on my entire well-being”*  *“…meditate at least 5 minutes every day”* |
| ***Sleeping behavior goals:***  Establish defined sleep hours, specific times for going to bed and waking up, or aim to create sleep routines (n=14) | *”…sleep at least 8 hours regularly”*  *”…go to bed at 21.30”*  *”…wake up before 08.00 every day”* |
| ***Physical body goals:***  Encompasses goals related to the physical body such as setting specific weight targets, aiming to improve physical well-being or committing to daily actions that are beneficial for one´s body (n=10) | *”…lose weight to 99 kg”*  *”…feel better physically”*  *”…do something that is good for my body every day”*  *”…to only drink coffee every other day (Monday, Wednesday, Friday)”* |
| ***Mobile phone behavior goals:***  Aiming to manage mobile phone usage, such as setting screen time limits or aiming to substitute phone usage with other activities (n=4) | *”…decrease my screen time”*  *“…use my mobile phone for a maximum of 2 hours per day”*  *“…read a book at night instead of watching videos”* |
| **Strategies to achieve miscellaneous behavior change (n=72)** | **In the upcoming week, I will undertake these two actions**  **to progress towards my goals…** |
| ***Sleep routines:*** Involves establishing sleep routines, with include maintaining the same bedtime- and wake up times each day, setting an alarm to initiate winding down in the evening, incorporating screen time restrictions, and ensuring timely waking up the next day (n=21) | *”…to be in bed by 21.30 and not use the mobile in bed, but read a book instead”*  *”…to go to bed on time and not sleep too late during the day”*  *”…to unwind before bedtime and to get up on time”*  *”…to get up on time and to put away or turn off the mobile”* |
| ***Self-reflection:*** Involves prioritizing self-care and planning to find time for rest, recovery, and reflection on one´s thoughts and feelings (n=14) | *”…to prioritize yourself first and remember that energy is not infinite”*  *”…to listen inwardly and to take it easy daily”* |
| ***Physical activity:*** Defines the type and duration of physical activity, along with planning for recovery, and consuming healthy food (n=13) | *”…by getting out on a morning walk and by preparing meal boxes (always having ready-made food in the fridge)”* |
| ***Task-orientation:*** Dedication to complete responsibilities such as academic assignments, cleaning, economy or other tasks (n=11) | *”…to vacuum, scrub the bathroom and tidy up everything that is lying around”*  *”…to refrain from small purchases and resist online shopping”* |
| ***Mobile phone discipline:*** Awareness and commitment to self-improve in studying and concentration by reducing mobile phone usage (n=8) | *“…to take regular breaks and to keep my phone in another room when I study”*  *”…by setting a time limit on my phone and not swipe it away”* |
| ***Making plans:*** Involves creating plans, setting up favorable conditions for oneself, staying focus and sticking to the plan (n=5) | *”…by scheduling study time every morning and getting up on time to be able to utilize all morning”* |
| **Motivational self-talk to encourage miscellaneous behavior change (n=72)** | **This is how I will remind and encourage myself when motivation wavers.**  **If it feels tough, I will tell myself that…** |
| ***Health benefits and sustainability:*** Self-encouragement and reminders about personal well-being benefits derived from increased physical activity, better sleep, reduced screen time, and completed tasks (n=24) | *”…I will feel good from exercise and less screen time. It will help”*  *”…I will feel so good in a clean apartment, I´ll feel so proud when it´s done”*  *“…I will feel so much better tomorrow and be able to concentrate better”* |
| ***Self-encouragement*:** Demonstrates empathy for one´s situation while simultaneously cheering oneself on and reminding oneself of their inherent capabilities, past successes, and the feasibility of task (n=23) | *”…I know it´s hard. But I rather do my best than give up anyway!”*  *”…come on, it´s only an hour”*  *”…you can do it, you are goal-oriented and wonderful”* |
| ***Time-management:*** Encouraging oneself to focus and actively prioritize and accomplish tasks to free up time for other aspects of life (n=12) | *”…when I do what I should, I get time over for other things, which I otherwise wouldn´t have time for”* |
| ***Self-value:*** Reminder of one´s inherent worth and self-love, choosing to prioritize personal well-being and health in pursuit of balance (n=10) | *”…my health is the most important thing, everything else will follow”*  *”…only you can love yourself the way you want to”* |
| I don´t know (n=3) | *”…I don´t know”* |
| **Rewards to treat oneself with after accomplishing miscellaneous behavior goals (n=72)** | **When I have achieved my goal, I will reward myself with…** |
| ***Shopping:*** Buy things for oneself such as clothing, shoes, beauty products, massage, home spa, facial treatment, home décor (n=20) | *”…something nice for my new apartment”*  *”…saving ¼ of my money for a perfume that I can afford after 1 month”* |
| ***Self-satisfaction:*** Give oneself an applause, a pat on the back, a hug, to feel pride of oneself and acknowledge one´s progress and effort (n=15) | *”…time to acknowledge my progress and treat myself with something small”*  *”…a hug and say good job!”* |
| ***Indulgence:*** Treat oneself to something tasty to eat, such as fika, ice-cream, chocolate, fruit-salad (n=11) | *”…a chocolate ball”*  *”…a nice fruit-salad on my balcony”* |
| ***Relaxation:*** Indulge in relaxing activities such as quiet time to relax, read a book or watch tv (n=9) | *”…guiltfree time with a book”*  *”…time to relax afterwards”* |
| ***Socializing:*** Time to hang out with friends and loved ones, but also giving oneself allowance to ask others for help (n=7) | *“…more time to hang out with others”*  *“…to eat a nice breakfast with [Name], either going out or at home”* |
| ***Nothing:*** I don´t need anything (n=5) | *“…nothing is needed…a kick-ass workout at the gym maybe”* |
| ***Economy:*** Money to save or invest, and the relief of not having to worry about losing my study grants (n=3) | *“…money to save up for future dreams”*  *“…I will not have to worry about losing my financial study allowance”* |
| ***Travel:*** Plans to go on a trip (n=2) | *“…planning and scheduling a day trip to Copenhagen”* |

| **Multiple health behavior goals (n=53)** | **In the upcoming week, my goal is to…** |
| --- | --- |
| ***Diet & Physical activity goals:***  Increase fruit and vegetable consumption, eat healthily, avoid unhealthy products, and become more physically active (n=22) | *“…consume 500 grams of vegetables daily and work out 5 times”*  *“…eat healthily and exercise 3 times”*  *”…say no when offered sweets, more physical activity”* |
| ***Health behaviors in combinations of three or more:***  Improve diet and physical activity, increase water intake, ensure adequate sleep, abstain from alcohol, reduce smoking, read more, practice meditation and yoga, lose weight, focus on studies, and accomplish tasks (n=10) | *”…maintain exercise and eating habits, and to sleep well”*  *”…balance school, diet, and exercise”*  *”…not drink (alcohol) at all, walk for 1.5 hours daily, and eat well”*  *”…meditate, exercise twice, attend meeting, and abstain from alcohol”* |
| ***Physical activity & Alcohol goals:***  Abstain from or limit consumption of alcohol and maintain physical activity for example through exercise, dancing or walking (n=9) | *”…exercise at least 3 times, no alcohol”*  *”…take at least 2 walks, at least 2 beer-free-days”*  *”…exercise at least 1 hour/day and not drink more than 7 units”* |
| ***Physical activity & Smoking goals:***  To be smoke-free or limit smoking to a specified maximum number of cigarettes, and to engage in walking or working out (n=6) | *”…not smoke, exercise 4 times a week”*  *“…stay smoke-free, to start exercising”* |
| ***Physical activity & Other routine goals:***  Increase physical activity by going outside, walking, or dancing and to accomplish study-related task and to engage in daily meditation (n=3) | *”…study and to be physically active for at least 60 minutes each day”*  *”…dance twice, to meditate daily and during study breaks”* |
| ***Alcohol, Smoking, Diet goals:***  Abstain from alcohol, or a limited consumption, to be smoke-free, along with diet goals (n=3) | *”…not drink any alcohol or smoke”*  *“…consume a maximum of 5 units this week and 3 candy-days”*  *“…not smoke and to eat more vegetables”* |
| **Strategies to achieve multiple health behavior goals (n=53)** | **In the upcoming week, I will undertake these two actions**  **to progress towards my goals…** |
| ***Restrictions:*** Establish limits on the consumption of alcohol, tobacco, and sweets, as well as making plans to be more physically active (n=17) | *”…schedule training and say no to cigarettes”*  *”…not go to a bar and walk 2 x 15 minutes”*  *”…schedule two training sessions and avoid hanging out with the wrong people”*  *”…not buy sweets when I go shopping and don´t drink alcohol every day”* |
| ***Routines:*** Incorporate more fruits and vegetables into their diet, increase water intake, schedule physical activity, and studying at specific times throughout the day (n=16) | *”…make salads and go for a vigorous walk/run (2 km) in the morning”*  *”…drink water more often, walk around my neighborhood or exercise at home”*  *”…take a walk after studying and start studying no later than 9 am every day”* |
| ***Preparations:*** Activities that enables healthy eating and physical activity, such as adding fruits and vegetables to the grocery shopping list, meal preparations and making lunchboxes (n=11) | *”…prepare clothes for walking/training and buy tasty fruit”*  *”…plan grocery shopping, cooking, and exercise early in the morning”*  *”…buy vegetables and make food boxes”* |
| ***Prioritization:*** Choose to focus on what is deemed important for achieving one´s goal, be persistent and resist avoidance behavior (n=6) | *”…prioritize my needs above all else, and set reasonable sub-goals in my studies”*  *”…create daily schedules and keep my phone in different room while studying”*  *”…avoid skipping training and plan my meals”* |
| ***Recovery:*** Ensure adequate recovery by setting aside personal time, sleeping sufficiently, and enjoying good music (n=3) | *”…take some me-time in the evenings and always keep a salad ready in the fridge”*  *“…catch enough sleep and listen to good music"* |
| **Motivational self-talk to encourage multiple health behavior change (n=53)** | **This is how I will remind myself when motivation diminishes.**  **If it becomes challenging, I will remind myself that…** |
| ***Health benefits:*** Involves reminding oneself that changing their behavior will result in feeling better and more energetic - subsequently. That good health is essential to cope, and that smoking isn´t worth it and that there are other alternatives than alcohol (n=24) | *”…I feel so much better from it”*  *”…this will bring me energy”*  *”…one can have fun without wine in the glass constantly, tomorrow will be good”*  *”…nope, it´s not worth it, I want to live a long life, I want to have a healthy body”* |
| ***Self-persuasion and encouragement:*** Reminding oneself of one´s capability and determination ‘*I can and I will’*, acknowledge past accomplishments and challenges overcome, while recognizing the necessity of effort in achieving goals and keeping promises to oneself (n=15) | *”…come on, you want this, you´ve done it before!”*  *”…remember how far you´ve come”*  *”…aiming to be a superhero, even if no one praises you for it – one day at a time”*  *”…you must keep your promises to yourself so that you can trust yourself”* |
| ***Goal-orientation:*** Reminding oneself to focus on the goal, it´s significance and why this is important. Acknowledging that one´s actions are undertaken to accomplish self-improvement (n=14) | *”…you can do it, think of the goal”*  *”…remember why we are doing this, becoming the best version of myself”*  *”…you are doing this for you”* |
| **Rewards to treat oneself with after accomplishing multiple health behavior goals (n=53)** | **When I have achieved my goal, I will reward myself with…** |
| ***Shopping:*** Buy myself a gift such as a new tablet, a phone, clothing, a bike, a book, nail varnish, coffee or lottery tickets (n=13) | *”…buy something nice for myself, coffee or book, whatever”*  *”…a new dress”*  *”…treat myself to something nice”* |
| ***Indulgence:*** Involves treating oneself to something tasty, such as a nice dinner or take-away-food, or sweets, crisps, or ice-cream (n=11) | *”…something tasty but cheap like crisps, soda and sweets, as a treat”*  *”…permit myself to buy take-way food and sweets”* |
| ***Relaxation:*** Indulge in relaxing activities such as arranging at-home-spa including a bath and a face mask, taking a day off, resting, sleeping, and meditating (n=10) | *”…meditation”*  *”…proper day´s rest with a fika”*  *”…at-home spa-day with a face mask, good wine and a plate of fruit”* |
| ***Self-satisfaction:*** Give oneself a pat on the back or a hug, to experience satisfaction with oneself, to feel proud and grateful for the affirmation of success, and to have a clear conscience (n=10) | *”…then I will be pleased with myself”*  *”…the validation of having accomplished my goal”*  *”…being extra grateful to myself for having accomplished what I set out to do”* |
| ***Enjoyment:*** Activities one enjoys doing such as having fun, socializing with good company, enjoying a dinner- or move-night, taking a trip, or visiting to the library (n=8) | *“…eating out with my partner”*  *“…every week I accomplish my goal I will reward myself with 100 SEK to save for something fun”* |
| I don´t know (n=1) | *“…I don´t know”* |

| **Alcohol consumption goals (n=19)** | **In the upcoming week, my goal is to…** |
| --- | --- |
| ***Amount limits:*** 'Not more than', 'maximum units', or 'less than' (n=14) | *” …not drink more than 6 units a week”* |
| ***Restricted drinking:***  To not drink at all, or during a specific time frame (n=5) | *”…not drink alcohol on weekdays”* |
| Unspecified limit, 'just less' (n=1) | *”…to drink less”* |
| **Strategies to achieve alcohol consumption behavior change (n=19)** | **In the upcoming week, I will undertake these two actions**  **to progress towards my goals…** |
| ***Replacements:*** Involves swapping alcohol for alternatives such as soda or alcohol-free options. It also includes substituting alcohol-related activities like going out, with activities that do not involve alcohol such as calling a friend, studying, or exercising. (n=8) | *”…practice saying no and drinking soda instead”*  *”…make some plans for Saturday night instead of going to the pub”.*  *“…avoid going to dinners, and exercise instead of staying on the sofa”* |
| ***Adjust drinking habits:*** Includes making modifications (to one´s drinking habits), such as slowing down the pace of drinking, abstaining from drinking before going out, planning to leave parties earlier, maintaining awareness meanwhile drinking, setting limits on the number of drinks or designated drinking days (n=7) | *”…drink slowly and leave earlier”*  *”…not drinking at home before the party,*  *and maintaining awareness during the pub crawl”*  *”… abstain from alcohol on Monday, Tuesday, and Thursday, and limit consumption to a maximum of 5 units over the weekend”* |
| ***Availability:*** Limiting access to alcohol by either not purchasing it at all or buying less, or setting boundaries for oneself when serving alcohol, asking for support, and adding activities such as taking a walk (n=4) | *”…to buy less alcohol”*  *”…avoiding shopping for alcohol and walking at least 30 minutes each day”*  *”…inform [Name] and pour a suitable amount into a carafe”* |
| **Motivational self-talk to encourage alcohol consumption behavior change (n=19)** | **This is how I will remind myself when motivation diminishes.**  **If it becomes challenging, I will remind myself that…** |
| ***Well-being and health benefits:*** Involves reminding oneself that reducing alcohol consumption can lead to improved well-being the following day, and avoidance of negative health consequences such as tiredness, anxiety or insomnia (n=12) | *”…I feel much better during the week if I don´t drink”*  *”…I gain better control and feel good the next day”*  *”…I avoid anxiety the next day if I drink moderately”* |
| ***Self-control:*** Entails reinforcing one´s belief in their capability to abstain from alcohol “you can do it”, and a reminder that sobriety enhances one´s learning capabilities (n=6) | *”…I can manage not to drink alcohol for a month”*  *”…I want to be able to handle alcohol and feel more energetic”*  *”…I learn better when I´m sober, I don´t want to embarrass myself”* |
| Unspecified (n=1) | *”…that the dog wants to go for a walk”* |
| **Rewards to treat oneself with after accomplishing alcohol consumption behavior goals (n=19)** | **When I have achieved my goal, I will reward myself with…** |
| ***Shopping:*** Treat myself with something like clothing, a bag, a phone, a face mask, a flower, home décor, or something tasty like tea or a poke bowl (n=10) | *”…buying a fancy face mask”*  *”…treating myself with a tasty poke bowl”*  *”…buy myself a new piece of clothing”* |
| ***Feelgood:*** Includes the reward of improved well-being, better sleep, reduced anxiety, recognition through a pat on the back, and financial savings from money not spent on alcohol (n=6) | *“…a pat on the back”*  *“…a better sleep and not having to deal with anxiety”*  *“…saving the money”* |
| ***Enjoyments:*** Engage in things I enjoy like taking a day off, booking a padel game, or using the money I´ve saved to go on a trip (n=3) | *”…a day off from studying”*  *”…invest my money in a trip”* |

| **Tobacco behavior goals (n=17)** | **In the upcoming week, my goal is to…** |
| --- | --- |
| ***Smoking goals:***  Defined amount and time limits (n=12) | *“… not smoke more than 4 cigarettes a day”*  *“…avoid smoking before 18.00”* |
| ***Smoking goals:***  Involves cutting down, to quit or to not smoke at all (n=3) | *“… reduce my smoking”*  *“…quit smoking”* |
| ***Snuff goals:***  Involves consumption limits and reducing nicotine strength (n=2) | *“…buy a maximum of 1 box of snuff”*  *“…quit snus by gradually reducing the nicotine strength monthly”* |
| **Strategies to achieve tobacco behavior change (n=17)** | **In the upcoming week, I will undertake these two actions**  **to progress towards my goal…** |
| ***Restrictions and awareness:*** Involves actions to limit access to tobacco (such as refraining from purchasing or carrying cigarettes or snuff) delaying smoking through time or quantity restrictions, resisting cravings, or making changes in the environment (n=9) | *“…stop buying cigarettes and drink water instead”*  *“…avoid smoking before lunch and after 18.00”*  *“…only keep 15 cigarettes in the pack and spend my day studying”*  *“…only bring 1 cigarette with me and avoid making my smoking area cozy”* |
| ***Substitutes:*** Involves replacing tobacco with alternatives such as drinking water, tea, chewing gum, eating, using nicotine replacement therapies, or engaging in relaxation exercises (n=4) | *“…resist the urge to smoke and to drink water instead”*  *“…chew gum, drink tea, and do breathing exercises”* |
| Unspecified (n=4) | *“…not smoke and not smoke”* |
| **Motivational self-talk to encourage tobacco behavior change (n=17)** | **This is how I will remind myself when motivation diminishes.**  **If it becomes challenging, I will remind myself…** |
| ***Health benefits:***  Involves reminding oneself that tobacco is not beneficial, and that one will feel better, be better off, and become healthier without it (n=10) | *“…that smoking is harmful to me and my asthma will improve without it”*  *“…to think about it long-term, I know my health will improve by quitting”* |
| ***Self-encouragement:***  Involves uplifting oneself, reminding oneself to persevere, striving to make oneself proud, and recalling previous accomplishments (n=6) | *“…that you can do this, come on now [Name]”*  *“…that you will be feeling very good when you have achieved your goal this week”*  *“…that you cannot afford it, and that I have succeeded in several demanding challenges before”* |
| Unspecified (n=1) | *“I don´t know”* |
| **Rewards to treat oneself with after accomplishing tobacco behavior goals (n=17)** | **When I have achieved my goal, I will reward myself with…** |
| ***Shopping:***  Buying things I want such as a phone, headphones, or activities like visiting the hairdresser or a nail salon (n=6) | *“…then I´ll buy something I´ve wanted for a long time”*  *“…a gift”*  *“…a new phone”* |
| ***Enjoyment and Self-satisfaction:***  Activities I enjoy doing such as playing computer games, self-satisfaction, and money for savings (n=4) | *“…gaming time at my computer”*  *“…the money I´ve saved”*  *“…a pat on the back”* |
| ***Smoking:*** To be allowed to smoke (n=4) | *“… a cigarette”* |
| ***Indulgence:*** Something tasty such as cheeses, prawns or sushi (n=2) | *“…then I´ll reward myself with sushi”* |
| I don´t know (n=1) | *“I don´t know”* |

| Self-authored challenges (n=170) |  |
| --- | --- |
| **Physical activity (n=60)** | **Examples of self-authored challenges** |
| ***Number of training days per week:***  Involves a defined training frequency ranging from 1-4 training days per week. The activities include working out, going to the gym, walking, running, swimming, boxing, strength exercises, stretching, yoga, and gardening work (n=30) | *”Go swimming 1 time during the week”*  *“I´m going to work out for 30 minutes twice this week”*  *”Running at least 20 minutes 3 times a week”*  *”I´m going to walk 10 000 steps a day for 4 days”* |
| ***Amount of training per day:***  Involves a defined training frequency per day including activities like a workout program, walking, running, yoga, squats, rowing machine and general movement (n=16) | *“Do a workout program for 10 minutes per day”*  *“I will run every weekday morning this week”*  *“I´ll try to walk 10 000 steps a day this week”* |
| ***Specified type of activity:***  Involves a diverse range of initiatives to be physically active including general movement, attending training sessions, gym workouts, yoga, running, obstacle courses, using a cross trainer, initiating fitness challenges, finding home workouts, and keeping track of one´s activity levels (n=14) | *“Go to the gym regularly”*  *“Move for 15 minutes after lunch”*  *“Find simple exercises to do at home”*  *“I will use the cross trainer or do brisk walking with poles“* |
| **Dietary behavior (n=30)** |  |
| ***Eat regularly and eat more vegetables:***  Involves planning to eat more regularly throughout the day, to plan and prepare meals and lunchboxes, to incorporate more vegetables, to eat a snack in the afternoon, and drink water (n=23) | *“Eat breakfast, lunch, dinner every day”*  *“Will plan meals this week”*  *“Eat salad with every meal”* |
| ***Reducing snacking:***  Involves avoiding from snacks, sugar, sweets, candy, energy drinks, take-away food, mindless snacking, and bread, or restrictions to limit the sugar intake to the equivalent of one pastry per day, or to cut down on treats and sweets but allow occasional indulgence (n=19) | *“I will not buy loose candy”*  *“I will not drink energy drinks”*  *“No snacks, fast food, or candy on weekdays”*  *“Cut down on fika and sweets, but treat yourself occasionally”* |
| **Mental health (n=19)** |  |
| ***Stress-reduction, meditation, reflection, gratitude and self-compassion:***  Involves activities aimed at reducing stress, enhancing mindfulness and meditation practices, improving journaling and reflective practices, and fostering gratitude and self-compassion (n=19) | *“I will practice mindfulness every day”*  *“Write down 3 positive things every evening”*  *“Breathe and remember that you can rely on yourself”*  *“Relax. Quit stressing. Take deep breaths!”* |
| **Tobacco use (n=14)** |  |
| ***Reduce and refrain from cigarettes and snus:***  Involves abstaining from purchasing or using tobacco before a specified time or hour, as well as aiming to reduce snuff consumption or decrease nicotine strength (n=14) | *“No smoking before 18.00”*  *“Maximum 1 snus every 2 hours”*  *“Do not smoke cigarettes until March”*  *“I will reduce the strength to 1 dot on the snus”* |
| **Study performance behavior (n=12)** |  |
| ***Time and task commitment for completion:***  Involves the intention to allocate time for specific tasks on a daily or weekly basis, as well as the commitment to complete study-related task including assignments (n=12) | *“Not postpone schoolwork”*  *“Complete math studies”*  *“You should dedicate the majority of your day for studying”*  *“To study for at least 5 hours per day this week”* |
| **Sleeping routines (n=8)** |  |
| ***Establishing sleep routines:***  Involves prioritizing sleep quality by establishing a sleep schedule, ensuring sufficient sleep each night, allowing time to unwind before bedtime, and going to bed and waking up on specific times (n=8) | *“I will follow my sleep schedule, bedtime at 21.30, wake up at 6”*  *“Go to bed before 01.00 every day”*  *“I will try to sleep as much as a can each night”* |
| **Leisure activities (n=6)** |  |
| ***Prioritize leisure activities:***  Involves the intention to allocate time for leisure activities such as gardening, reading more books, and being socially active, as well as the intention to spend less time on social media (n=5) | *“I will not check social media before lunch”*  *“Read more books and audio books”*  *“Be more social”* |
| **Alcohol consumption (n=5)** |  |
| ***Restrictions on consumption:***  Involves restrictions on maximum number of units of alcohol per week, number of consumption days, or total number of units (n=5) | *“I will drink a maximum of one glass of alcohol this week”*  *“I will only drink on Friday and Saturday between 19.00-22.00”* |
| **Physical health and wellbeing (n=4)** |  |
| Involves intentions that cover various aspects of physical health and well-being such as brushing teeth, taking medication and aiming for weight-loss (n=4) | *“Brush my teeth twice a day”*  *“Lose weight”* |
